# Dysregulation of innate and adaptive lymphoid immunity may have implications for symptom attribution and predict responses to targeted therapies in neuropsychiatric systemic lupus erythematosus

**DOI:** 10.1016/j.jtauto.2025.100296

**Published:** 2025-06-11

**Authors:** Julius Lindblom, Guillermo Barturen, Lorenzo Beretta, Daniel Toro-Domínguez, Elena Carnero-Montoro, Maria Orietta Borghi, Jessica Castillo, Ellen Iacobaeus, Yvonne Enman, PRECISESADS Clinical Consortium, Jacques-Olivier Pers, Alain Saraux, Valérie Devauchelle-Pensec, Sandrine Jousse-Joulin, Bernard Lauwerys, Julie Ducreux, Anne-Lise Maudoux, Ana Tavares, Isabel Almeida, Miguel Angel Gonzalez-Gay Mantecón, Ricardo Blanco Alonso, Alfonso Corrales Martínez, Ignasi Rodríguez-Pintó, Gerard Espinosa, Rik Lories, Nicolas Hunzelmann, Doreen Belz, Niklas Baerlecken, Michael Zauner, Michaela Lehner, Eduardo Collantes, Ma Angeles Aguirre-Zamorano, Alejandro Escudero-Contreras, Ma Carmen Castro-Villegas, Norberto Ortego, María Concepción Fernández Roldán, Enrique Raya, Inmaculada Jiménez Moleón, Enrique de Ramon, Isabel Díaz Quintero, Pier Luigi Meroni, Tommaso Schioppo, Carolina Artusi, Carlo Chizzolini, Aleksandra Zuber, Donatienne Wynar, Attila Balog, Magdolna Deák, Márta Bocskai, Sonja Dulic, Gabriella Kádár, Falk Hiepe, Silvia Thiel, Manuel Rodriguez Maresca, Antonio López-Berrio, Rocío Aguilar-Quesada, Héctor Navarro-Linares, Chandra Mohan, Marta E. Alarcón-Riquelme, Dionysis Nikolopoulos, Ioannis Parodis

**Affiliations:** l Centre Hospitalier Universitaire de Brest, Hospital de la Cavale Blanche, Brest, France; m Pôle de pathologies rhumatismales systémiques et inflammatoires, Institut de Recherche Expérimentale et Clinique, Université catholique de Louvain, Brussels, Belgium; n Centro Hospitalar do Porto, Portugal; o Servicio Cantabro de Salud, Hospital Universitario Marqués de Valdecilla, Santander, Spain; p Department of Autoimmune Diseases, Hospital Clínic, Institut d’Investigacions Biomèdiques August Pi i Sunyer, Barcelona, Catalonia, Spain; q Katholieke Universiteit Leuven, Belgium; r Klinikum der Universitaet zu Koeln, Cologne, Germany; s Medizinische Hochschule Hannover, Germany; t Medical University Vienna, Vienna, Austria; u Servicio Andaluz de Salud, Hospital Universitario Reina Sofía Córdoba, Spain; v Servicio Andaluz de Salud, Complejo hospitalario Universitario de Granada (Hospital Universitario San Cecilio), Spain; w Servicio Andaluz de Salud, Complejo hospitalario Universitario de Granada (Hospital Virgen de las Nieves), Spain; x Servicio Andaluz de Salud, Hospital Regional Universitario de Málaga, Spain; y Università degli studi di Milano, Milan, Italy; z Hospitaux Universitaires de Genève, Switzerland; aa University of Szeged, Szeged, Hungary; ab Charite, Berlin, Germany; ac Andalusian Public Health System Biobank, Granada, Spain; a Division of Rheumatology, Department of Medicine Solna, Karolinska Institutet, and Karolinska University Hospital, SE-17176, Stockholm, Sweden; b Center for Molecular Medicine, Stockholm, Sweden; c GENYO, Centre for Genomics and Oncological Research: Pfizer, University of Granada / Andalusian Regional Government, Medical Genomics, Granada, Spain; d Department of Genetics, Faculty of Sciences, University of Granada, Granada, Spain; e Referral Center for Systemic Autoimmune Diseases, Fondazione IRCCS Ca’ Granda Ospedale Maggiore Policlinico di Milano, Italy; f Department of Clinical Sciences and Community Health, Università Degli Studi di Milano, Milan, Italy; g IRCCS, Istituto Auxologico Italiano, Milan, Italy; h Department of Biomedical Engineering, University of Houston, Houston, TX, USA; i Neuroimmunology Unit, Department of Clinical Neuroscience, Karolinska Institutet, Stockholm, Sweden; j Institute of Environmental Medicine, Karolinska Institutet, Stockholm, Sweden; k Department of Rheumatology, Faculty of Medicine and Health, Örebro University, Örebro, Sweden

**Keywords:** Systemic lupus erythematosus, Neuropsychiatric systemic lupus erythematosus, Precision medicine, Druggability, Biologics, Transcriptome, Gene expression

## Abstract

**Objectives:**

To gain insights into the pathogenesis of neuropsychiatric systemic lupus erythematosus (NPSLE) and identify potential drug targets through investigation of whole-blood human transcriptome.

**Methods:**

We analysed differentially expressed genes in peripheral blood from active central nervous system (CNS) lupus (n = 26) and active non-neuropsychiatric SLE (n = 38) patients versus healthy controls (n = 497) from the European PRECISESADS project (NTC02890121). We further explored dysregulated gene modules in active CNS lupus and their correlation with serological markers. Lastly, we performed regulatory network and druggability analysis.

**Results:**

Unsupervised weighted gene co-expression network analysis (WGCNA) revealed 23 dysregulated gene modules and two subgroups of active CNS lupus. The interferon gene module was prominently upregulated in subgroup 1, while the B cell, T cell, and cytotoxic/natural killer (NK) cell modules were downregulated. Subgroup 2 showed less marked dysregulation patterns. Subgroup 1 had lower estimated proportions of lymphoid cell subsets and proportionally more patients positive for anti-dsDNA antibodies compared to subgroup 2, pointing to molecularly distinct subgroups or misclassification of subgroup 2. *In silico* prediction algorithms demonstrated a greater anticipated response to anifrolumab, C3 inhibitors, and calcineurin inhibitors for patients in CNS lupus subgroup 1 compared with subgroup 2.

**Conclusions:**

Gene dysregulation patterns related to innate and adaptive lymphoid immunity separated active CNS lupus patients into two distinct subgroups with differential anticipated response to type I interferon, C3, and calcineurin inhibition. Our study provides a conceptual framework for precision medicine in NPSLE and implications for overcoming the major clinical challenge of attributing neuropsychiatric features to SLE versus other causes.

## 1 Introduction

Neuropsychiatric systemic lupus erythematosus (NPSLE) is a common manifestation of systemic lupus erythematosus (SLE) affecting up to 20 % of the patients [1,2]. Mortality among patients with NPSLE is estimated at 16 % within 10 years from diagnosis. Despite complete resolution of NPSLE activity in several cases and low frequency of NPSLE flares, neuropsychiatric syndromes are associated with long-term neurological disability [3]. Importantly, NPSLE is associated with substantial impairments of patients’ health-related quality of life [4] and overall damage accrual [5]. Hence, better management of NPSLE is an urgent need.

The current management of NPSLE is empirical and extrapolated from extra-neurological manifestations of SLE, or from non-SLE neurological conditions, mainly due to the lack of clinical trials [6,7]. To date, only one randomised controlled trial (RCT) has been successful for severe NPSLE, which demonstrated a clinical benefit from intravenous cyclophosphamide [8,9]. The European Alliance of Associations for Rheumatology (EULAR) has endorsed eminence- and evidence-based recommendations for standardising and improving the care of SLE patients presenting with neuropsychiatric events [10]. However, these recommendations were based on low-grade evidence, highlighting the need for further research.

Research within NPSLE has been poor due to limited access to tissue, the heterogeneity of clinical syndromes, and the overlap with neuropsychiatric events which are not related to SLE imposing the known clinical challenge of correct attribution of symptoms [11,12]. Most current evidence is derived from animal and neuroimaging studies. To date, murine studies have led to only two clinical trials in NPSLE patients. One tested memantine but showed no benefit in improving cognitive performance [13]. The other was announced in 2019 and is testing captopril, with its primary outcomes involving hippocampal metabolism and microglial activation assessed using brain positron emission tomography (PET) [14]. Studies of translational nature using human samples that could facilitate hypothesis generation for drug development for NPSLE have been limited [15]. This need formed the aim of the present study, which was to investigate the transcriptome of patients with NPSLE to gain insights into underlying molecular mechanisms and identify potential drug targets.

## 2 Patients and methods

### 2.1 Study population

Peripheral blood samples and clinical information were collected from 350 patients with SLE, all meeting the revised American College of Rheumatology (ACR) criteria for SLE [16], as well as from 497 HC, within the frame of the 5-year European PRECISESADS project (NTC02890121) [17].

The complete set of inclusion and exclusion criteria is available in Supplementary Table S1. Active CNS lupus was defined as a score of 8 or more in the CNS descriptors of SLE Disease Activity Index 2000 (SLEDAI-2K) [18] and/or physician-reported CNS involvement according to the PRECISESADS case report form (CRF) (n = 26; see Supplementary Material, page 5 for a list of CNS syndromes). Active SLE with no neuropsychiatric history was defined as a score of 8 or more in the clinical version of SLEDAI-2K (cSLEDAI-2K) but zero score in the CNS descriptors and absence of current or past CNS involvement according to the PRECISESADS CRF (n = 38).

Before recruitment in PRECISESADS, all patients and HC provided informed consent. The PRECISESADS protocol received approval from local ethics review boards at all participating centres (see Supplementary Material, page 6 for a list of local investigators). The present study was approved by the Swedish Ethical Review Authority (registration number: 2022-03907-01).

### 2.2 Sample data

Genome-wide RNA sequencing of peripheral whole blood was performed using Illumina assays (Illumina Inc., San Diego, CA, USA), as previously detailed [17]. Serum levels of selected cytokines and autoantibodies were measured, following protocols described elsewhere [17]. Initially, a comprehensive analysis of 88 cytokines was conducted on a subset of patients and HC using Luminex xMAP Technology (Luminex Corporation, Austin, TX, USA). Subsequently, a custom panel from R&D Systems (Luminex assay, Luminex Corporation, Austin, TX, USA) was utilised to measure a subset of 14 cytokines, while 6 additional cytokines were analysed using a quantitative sandwich enzyme immunoassay from Biorad Laboratories Inc. (Hercules, CA, USA). Autoantibody levels were measured using an automated chemiluminescent immunoassay (IDS-iSYS, Immunodiagnostic Systems Holdings Ltd., East Boldon, United Kingdom), a turbidimetric immunoassay (SPAPLUS analyser, The Binding Site Group Ltd., Birmingham, United Kingdom), and an enzyme-linked immunosorbent assay (ELISA) kit from EUROIMMUN Medizinische Labordiagnostika AG (Lübeck, Germany).

### 2.3 Bioinformatic and statistical analysis

We first performed differential gene expression analysis in patients with active CNS lupus (n = 26) versus HC (n = 497), as well as in patients with active SLE but no history of NPSLE (n = 38) versus HC, adjusting for age, sex, sequencing batch, and RNA integrity number (RIN). Pathway enrichment analysis was performed by over-representation analysis (ORA). We identified gene modules of relevance to active CNS using weighted gene co-expression network analysis (WGCNA) based on full transcriptome data and assessed their dysregulation relative to age- and sex-matched HC at a 1:5 ratio (n = 130). Gene expression data were used to estimate the relative proportions of 22 immune cell types; as such, relative increases in the proportion of certain cell subsets result in decreases in other subsets due to the compositional nature of the data. Correlation analyses were performed between dysregulation scores of gene modules and serum levels of selected serological markers using Spearman's rank correlation coefficients, while the Mann-Whitney *U* test was used to assess gene dysregulation in relation to positivity for conventional autoantibodies, based on cut-offs as recommended by the assay manufacturer [17]. Dysregulated gene modules were assessed with regard to signalling molecule networks, followed by druggability analysis. A detailed description of the analytical pipeline is found in the Supplementary Material, page 7–9.

### 2.4 Patient and public involvement

A patient research partner (YE) was involved in the design and reporting of this research. The public was not involved in the design, or conduct, or reporting or dissemination plans of this research.

## 3 Results

Demographics and clinical data of patients and HC, all of whom were of White/Caucasian European descent, are presented in Table 1. The group with active CNS lupus had lower renal activity compared to the active non-neuropsychiatric SLE group (19 % versus 53 %; *p* = 0.015) whereas no significant differences were observed in other organ systems.

**Table 1:** Characteristics of patients with active CNS lupus, active non-neuropsychiatric SLE, and healthy controls from the PRECISESADS study population.

		Comparators		
	**Active CNS lupus**	**Active non-neuropsychiatric SLE**	**HC**	***p* active CNS lupus vs. active non-neuropsychiatric SLE**
	**n=26**	**n=38**	**n=497**	
Demographics				
Age (years); mean (s.d.)	45.5 (14.6)	48.0 (14.3)	47.1 (13.0)	0.498
Female sex; n (%)	25 (96.2)	35 (92.1)	393 (79.1)	0.895
White/Caucasian of European origin; n (%)	26 (100)	38 (100)	497 (100)	N/A
Clinical data				
Disease duration (years); mean (s.d.)	15.5 (9.9)	16.2 (10.8)	N/A	0.810
SLEDAI-2K score; mean (s.d.)	15.7 (7.2)	13.6 (4.1)	N/A	0.140
CNS; n (%)				
Seizure	2 (10.0); N = 20	0.0 (0.0); N = 37	N/A	0.229
Psychosis	3 (14.3); N = 21	0.0 (0.0)	N/A	0.076
Organic brain syndrome	2 (9.5); N = 21	0.0 (0.0)	N/A	0.236
Visual disturbance	6 (28.6); N = 21	0.0 (0.0)	N/A	0.002
Cranial nerve disorder	1 (4.8); N = 21	0.0 (0.0)	N/A	0.762
Lupus headache	7 (33.3); N = 21	0.0 (0.0)	N/A	**0.001**
CVA	3 (14.3); N = 21	0.0 (0.0)	N/A	0.076
Vascular; n (%)	3 (11.5)	11 (28.9)	N/A	0.178
Musculoskeletal; n (%)	5 (19.2)	17 (44.7)	N/A	0.065
Renal; n (%)	5 (19.2)	20 (52.6)	N/A	**0.015**
Dermal; n (%)	15 (57.7)	27 (71.1)	N/A	0.402
Serosal; n (%)	0 (0.0)	3 (7.9)	N/A	0.387
Immunologic; n (%)	16 (61.5)	31 (81.6)	N/A	0.135
Constitutional; n (%)	1 (3.8)	2 (5.3)	N/A	1.000
Haematologic; n (%)	4 (15.4)	9 (23.7)	N/A	0.621
Serological profile				
Anti-dsDNA (U/mL); median (IQR)	2.1 (0.0–30.6); N = 21	33.5 (2.2–102.4); N = 30	N/A	**0.046**
Anti-dsDNA (+; ≥40); n (%)	4 (19.0); N = 21	15 (50.0); N = 30	N/A	0.050
Anti-Sm (U/mL); median (IQR)	0.0 (0.0–0.0); N = 20	0.0 (0.0–0.0); N = 28	N/A	0.305
Anti-Sm (+; ≥10); n (%)	0 (0.0); N = 20	2 (7.1); N = 28	N/A	0.625
Anti-β_2_GPI IgG (U/mL); median (IQR)	0.0 (0.0–0.6); N = 21	0.0 (0.0–0.0); N = 29	N/A	0.061
Anti-β_2_GPI IgG (+; ≥20); n (%)	3 (14.3); N = 21	2 (6.9); N = 29	N/A	0.702
Anti-β_2_GPI IgM (U/mL); median (IQR)	0.0 (0.0–1.5); N = 21	0.0 (0.0–0.0); N = 30	N/A	0.159
Anti-β_2_GPI IgM (+; ≥20); n (%)	2 (9.5); N = 21	1 (3.4); N = 29	N/A	0.772
aCL IgG (U/mL); median (IQR)	0.0 (0.0–4.9); N = 21	0.0 (0.0–0.4); N = 30	N/A	0.341
aCL IgG (+; ≥20); n (%)	3 (14.3); N = 21	3 (10.0); N = 30	N/A	0.979
aCL IgM (U/mL); median (IQR)	0.0 (0.0–0.0); N = 21	0.00 (0.0–0.1); N = 30	N/A	0.960
aCL IgM (+; ≥20); n (%)	3 (14.3); N = 21	1 (3.3); N = 30	N/A	0.367
C3c (g/L); median (IQR)	1.1 (0.6–1.4); N = 21	0.8 (0.6–1.1); N = 30	N/A	0.275
Low C3c (normal range: 0.81–1.57); n (%)	7 (33.3); N = 21	15 (50.0); N = 30	N/A	0.371
C4 (g/L); median (IQR)	0.2 (0.1–0.3); N = 21	0.2 (0.1–0.2); N = 30	N/A	0.789
Low C4 (normal range: 0.13–0,39); n (%)	9 (42.9); N = 21	12 (40.0); N = 30	N/A	1.000
Medications (current use)				
Prednisone equivalent dose (mg/day); mean (s.d.)	3.5 (3.0); N = 18	4.1 (4.0); N = 36	N/A	0.585
Antimalarial agents; n (%)	18 (69.2)	28 (73.7)	N/A	0.915
Immunosuppressants; n (%)				
Azathioprine	2 (9.5); N = 21	8 (21.1)	N/A	0.443
Calcineurin inhibitors	0 (0.0); N = 21	1 (2.6)	N/A	1.000
Leflunomide	0 (0.0); N = 21	0 (0.0)	N/A	N/A
Methotrexate	4 (19.0); N = 21	3 (7.9)	N/A	0.396
Mycophenolic acid	1 (4.8); N = 21	12 (31.6)	N/A	**0.040**

Data are presented as the number (percentage) or mean ± standard deviation. In case of non-normal distributions, the median (interquartile range) is indicated. In case of missing values, the total number of patients with available data is indicated. Statistically significant *p* values are in bold.

aCL: antibodies against cardiolipin; anti-β_2_GPI: antibodies against β_2_-glycoprotein I; anti-dsDNA: antibodies against double-stranded DNA; anti-Sm: antibodies against Smith; C3c: complement component 3c; C4: complement component 4; CNS: central nervous system; HC: healthy controls; Ig: immunoglobulin; IQR: interquartile range; N/A: not applicable; s.d.: standard deviation; SLE: systemic lupus erythematosus; SLEDAI-2K: Systemic Lupus Erythematosus Disease Activity Index 2000.

### 3.1 Transcriptomic aberrancies in active CNS lupus

**Fig. 1:**
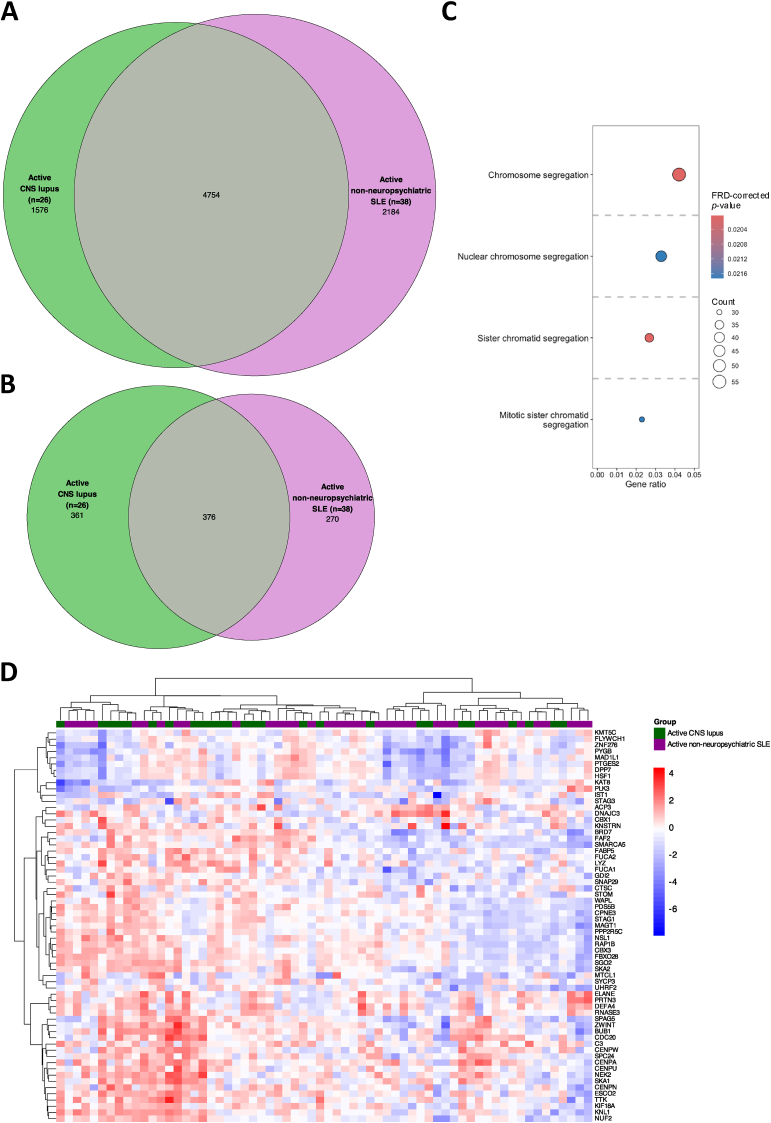
DEGs in patients with active CNS lupus and active non-neuropsychiatric SLE versus HC. **(A)** The Venn diagrams show DEGs (top) and **(B)** the subset of DEGs that exceeded the |log_2_ FC| >0.58 threshold (bottom) in patients with active CNS lupus (n = 26) versus HC (n = 497; in green), and in patients with active non-neuropsychiatric SLE (n = 38) versus HC (n = 497; in purple). **(C)** The enriched BP GO terms from over-representation analysis are plotted, based on CNS lupus-specific DEGs in patients with active CNS lupus versus HC (panel A, n = 1576). The size of the dots represents the gene count, and the gene ratio at the bottom of each dot plot represents the ratio between the gene count and the total number of genes included in the GO term. The colour of the dots corresponds to the FDR-corrected *p*-value from the pathway enrichment analysis. **(D)** The heatmap shows gene expression patterns in patients with active CNS lupus (green) or active non-neuropsychiatric SLE (purple) from the BP chromosome segregation GO term. Only DEGs that exceeded the |log_2_ FC| >0.58 threshold in the DEG analysis among patients with active CNS lupus versus HC are included in the heatmap. Columns denote SLE patients and rows denote CNS lupus-specific DEGs, measured by z-scores in relation to HC, clustered using hierarchical clustering with the Ward method. BP: biological process; CNS: central nervous system; DEGs: differentially expressed genes; FC: fold change; FDR: false discovery rate; GO: gene ontology; HC: healthy controls; SLE: systemic lupus erythematosus.

We identified 6330 DEGs in active CNS lupus (n = 26) compared to HC (n = 493). Among these, 4754 DEGs were shared between patients with active CNS lupus and patients with active SLE but no history of NPSLE (n = 38; Fig. 1A; detailed in Supplementary Material, sheets 1–2). Of the remaining 1576 CNS lupus-specific genes (Figs. 1A), 361 DEGs exhibited a |log_2_ fold change (FC)| >0.58 (Fig. 1B). Pathway enrichment analysis showed that the active CNS lupus gene signature encompassed DEGs related to chromosome segregation, among other pathways (Fig. 1C; detailed in Supplementary Material, sheets 3–6). Patients with active CNS lupus generally showed upregulation of the genes included in the enriched “Chromosome segregation” gene ontology (GO) term, where most genes from the active CNS lupus signature were identified (Fig. 1D; detailed in Supplementary Material, sheet 5). This GO term included the complement component 3 (*C3*), cell division cycle 20 (*CDC20*), proteinase 3 (*PRTN3*), and cathepsin C (*CTSC*) genes, underscoring the significance of inflammation and apoptosis in CNS lupus.

### 3.2 Dysregulated gene modules and molecular subgroups of active CNS lupus

**Fig. 2:**
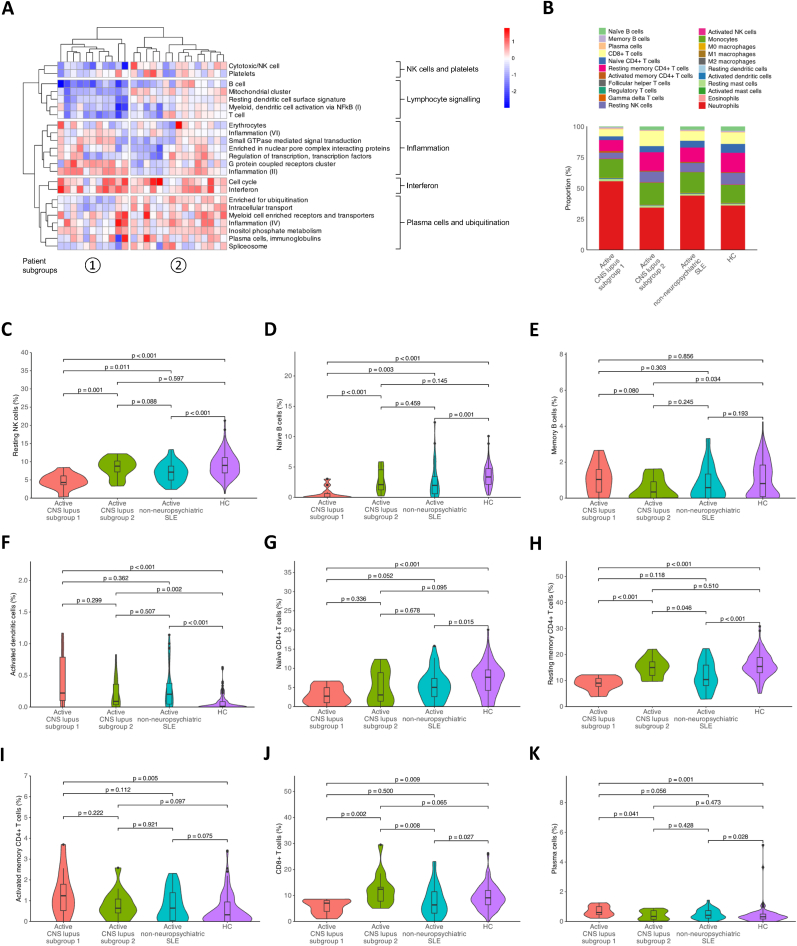
Dysregulated gene modules in patients with active CNS lupus. **(A)** The heatmap shows replicated gene modules and their dysregulation in relation to the gene expression of age- and sex-matched HC (n = 130), as measured by the z-score, in patients with CNS lupus (n = 26). Columns denote CNS lupus patients, and rows denote gene modules, clustered using hierarchical clustering with the Ward method. **(B)** The bar plot displays deconvolution results of estimated relative immune cell type proportions across active CNS lupus subgroups (n = 11 for subgroup 1 and n = 15 for subgroup 2), patients with active non-neuropsychiatric SLE (n = 38), and HC (n = 130). **(C**–**K)** Violin plots displaying distributions of estimated relative immune cell subset proportions across active CNS lupus subgroups, patients with active non-neuropsychiatric-SLE, and HC for **(C)** resting NK cells, **(D)** naïve B cells, **(E)** memory B cells, **(F)** activated dendritic cells, **(G)** naïve CD4^+^ T cells, **(H)** resting memory CD4^+^ T cells, **(I)** activated memory CD4^+^ T cells, **(J)** CD8^+^ T cells, and **(K)** plasma cells. Selected immune cell subsets of particular relevance are labelled, and comparisons for all immune cell subsets are provided in Supplementary Material, sheet 10. NK: natural killer; SLE: systemic lupus erythematosus.

Beyond DEG analysis, we examined dysregulated gene modules to identify co-expressed genes involved in shared biological processes and assess whether their dysregulation patterns could stratify patients with active CNS lupus. We identified 23 gene modules among the 26 active CNS lupus patients, of which eight were also identified in group with active SLE but no history of NPSLE, and four were contiguous (detailed in Supplementary Material, sheets 7–9). These 23 dysregulated gene modules formed five main clusters, which were manually annotated based on their compositions as NK cells and platelets, lymphocyte signalling, inflammation, interferon (IFN), and plasma cells and ubiquitination. The active CNS lupus patients were clustered into two distinct subgroups (Fig. 2A). Subgroup 1 (n = 11; 36 %) had significantly higher haematological activity compared to subgroup 2 (n = 15; 0 %; *p* = 0.047; Table 2). Four of nine patients with available data in subgroup 1 (44 %) but none among 12 patients in subgroup 2 were anti-dsDNA positive. Subgroup 1 was characterised by prominent upregulation of the IFN and inflammation (II) gene modules and downregulation of the B cell, T cell, and cytotoxic/NK cell gene modules compared with HC, while subgroup 2 showed less marked dysregulation. Similarly, dysregulation scores differed between the two patient subgroups for all aforementioned gene modules (*p* < 0.001 for all), with exception of the IFN gene module (*p* = 0.217; Supplementary Fig. S1 and Supplementary Table S2). The two patient subgroups did not differ in overall disease activity or CNS manifestations as measured by SLEDAI-2K and its CNS descriptors (Table 2). Further clustering within subgroup 2 revealed two distinct patient subsets, with one subset displaying greater downregulation of B cell and T cell gene modules, as well as gene modules within the inflammation cluster, compared with the other patient subset (Fig. 2A).

**Table 2:** Characteristics of subgroups of patients with active CNS lupus from the PRECISESADS study population.

	Active CNS lupus subgroups		*p*
	**1**	**2**	
	**n=11**	**n=15**	
Demographics			
Age (y); mean (SD)	43.7 (15.1)	46.8 (14.6)	0.550
Female sex; n (%)	11 (100.0)	14 (93.3)	1.000
White/Caucasian of European origin; n (%)	11 (100.0)	15 (100)	N/A
Clinical data			
Disease duration (y); mean (SD)	13.7 (6.7)	16.9 (11.8)	0.736
SLEDAI-2K score; mean (SD)	16.5 (5.7)	15.2 (8.4)	0.333
CNS; n (%)			
Seizure	2 (22.2); N = 9	0 (0.0); N = 11	0.369
Psychosis	0 (0.0); N = 9	3 (25.0); N = 12	0.322
Organic brain syndrome	0 (0.0); N = 9	2 (16.7); N = 12	0.592
Visual disturbance	2 (22.2); N = 9	4 (33.3); N = 12	0.944
Cranial nerve disorder	0 (0.0); N = 9	1 (8.3); N = 12	1.000
Lupus headache	2 (22.2); N = 9	5 (41.7); N = 12	0.640
CVA	2 (22.2); N = 9	1 (8.3); N = 12	0.787
Vascular; n (%)	2 (18.2)	1 (6.7)	0.774
Musculoskeletal; n (%)	3 (27.3)	2 (13.3)	0.698
Renal; n (%)	2 (18.2)	3 (20.0)	1.000
Dermal; n (%)	6 (54.5)	9 (60.0)	1.000
Serosal; n (%)	0 (0.0)	0 (0.0)	N/A
Immunologic; n (%)	8 (72.7)	8 (53.3)	0.551
Constitutional; n (%)	1 (9.1)	0 (0.0)	0.874
Haematologic; n (%)	4 (36.4)	0 (0.0)	**0.047**
Serological profile			
Anti-dsDNA (U/mL); median (IQR)	30.6 (0.0–68.7); N = 9	1.2 (0.0–10.7); N = 12	0.154
Anti-dsDNA (+; ≥40); n (%)	4 (44.4); N = 9	0 (0.0); N = 12	**0.045**
Anti-Sm (U/mL); median (IQR)	0.0 (0.0–0.0); N = 8	0.0 (0.0–0.0); N = 12	0.221
Anti-Sm (+; ≥10); n (%)	0 (0.0); N = 8	0 (0.0); N = 12	N/A
Anti-β_2_GPI IgG (U/mL); median (IQR)	0.0 (0.0–0.6); N = 9	0.0 (0.0–0.2); N = 12	0.374
Anti-β_2_GPI IgG (+; ≥20); n (%)	2 (22.2); N = 9	1 (8.3); N = 12	0.787
Anti-β_2_GPI IgM (U/mL); median (IQR)	0.0 (0.0–1.5); N = 9	0.0 (0.0–0.4); N = 12	0.624
Anti-β_2_GPI IgM (+; ≥20); n (%)	1 (11.1); N = 9	1 (8.3); N = 12	1.000
aCL IgG (U/mL); median (IQR)	0.2 (0.0–4.9); N = 9	0.0 (0.0–2.2); N = 12	0.386
aCL IgG (+; ≥20); n (%)	2 (22.2); N = 9	1 (8.3); N = 12	0.787
aCL IgM (U/mL); median (IQR)	0.0 (0.0–0.0); N = 9	0.0 (0.0–0.4); N = 12	0.887
aCL IgM (+; ≥20); n (%)	1 (11.1); N = 9	2 (16.7); N = 12	1.000
C3c (g/L); median (IQR)	1.0 (0.5–1.2); N = 9	1.2 (0.8–1.5); N = 12	0.256
Low C3c (normal range: 0.81–1.57); n (%)	4 (44.4); N = 9	3 (25.0); N = 12	0.640
C4 (g/L); median (IQR)	0.1 (0.1–0.2); N = 9	0.2 (0.2–0.3); N = 12	**0.047**
Low C4 (normal range: 0.13–0,39); n (%)	6 (66.7); N = 9	3 (25.0); N = 12	0.143
Medications (current use)			
Prednisone equivalent dose (mg/day); mean (SD)	4.5 ± 3.3; N = 8	2.7 ± 2.7; N = 10	0.173
Antimalarial agents; n (%)	8 (72.7)	10 (66.7)	1.000
Immunosuppressants; n (%)			
Azathioprine	1 (11.1); N = 9	1 (8.3); N = 12	1.000
Calcineurin inhibitors	0 (0.0); N = 9	0 (0.0); N = 12	N/A
Leflunomide	0 (0.0); N = 9	0 (0.0); N = 12	N/A
Methotrexate	2 (22.2); N = 9	2 (16.7); N = 12	1.000
Mycophenolic acid	1 (11.1); N = 9	0 (0.0); N = 12	0.882

Data are presented as the number (percentage) or mean ± standard deviation. In case of non-normal distributions, the median (interquartile range) is indicated. In case of missing values, the total number of patients with available data is indicated. Statistically significant *p* values are in bold.

aCL: antibodies against cardiolipin; anti-β_2_GPI: antibodies against β_2_-glycoprotein I; anti-dsDNA: antibodies against double-stranded DNA; anti-Sm: antibodies against Smith; C3c: complement component 3c; C4: complement component 4; CNS: central nervous system; HC: healthy controls; Ig: immunoglobulin; IQR: interquartile range; N/A: not applicable; s.d.: standard deviation; SLEDAI-2K: Systemic Lupus Erythematosus Disease Activity Index 2000.

### 3.3 Immune cell subsets in active CNS lupus

Cell deconvolution analysis revealed a relative abundance of myeloid cells in patients with active CNS lupus patients within subgroup 1 (n = 11), as indicated by a higher estimated neutrophil to lymphocyte ratio (NLR) in this subgroup than in patients within subgroup 2 (n = 15), in patients with active non-neuropsychiatric SLE patients (n = 38), and in matched HC (n = 130; Fig. 2B; Supplementary Table S3–S4). The active CNS lupus subgroup 1 also had lower estimated proportions of resting NK cells (Fig. 2C) and naïve B cells (Fig. 2D) but not memory B cells (Fig. 2E), activated dendritic cells (Fig. 2F), or naïve CD4^+^ T cells (Fig. 2G) compared with subgroup 2 and with patients with active non-neuropsychiatric SLE. Additionally, subgroup 1 was characterised by a relative abundance of resting memory CD4^+^ T cells (Fig. 2H) but not activated memory CD4^+^ T cells compared with subgroup 2 (Fig. 2I). Lastly, estimated CD8^+^ T cell proportions (Fig. 2J) were lower and plasma cell proportions were higher in subgroup 1 versus subgroup 2 (Fig. 2K; Supplementary Material, sheet 10).

### 3.4 Cytokine and autoantibody profiles in relation to dysregulated gene modules

**Fig. 3:**
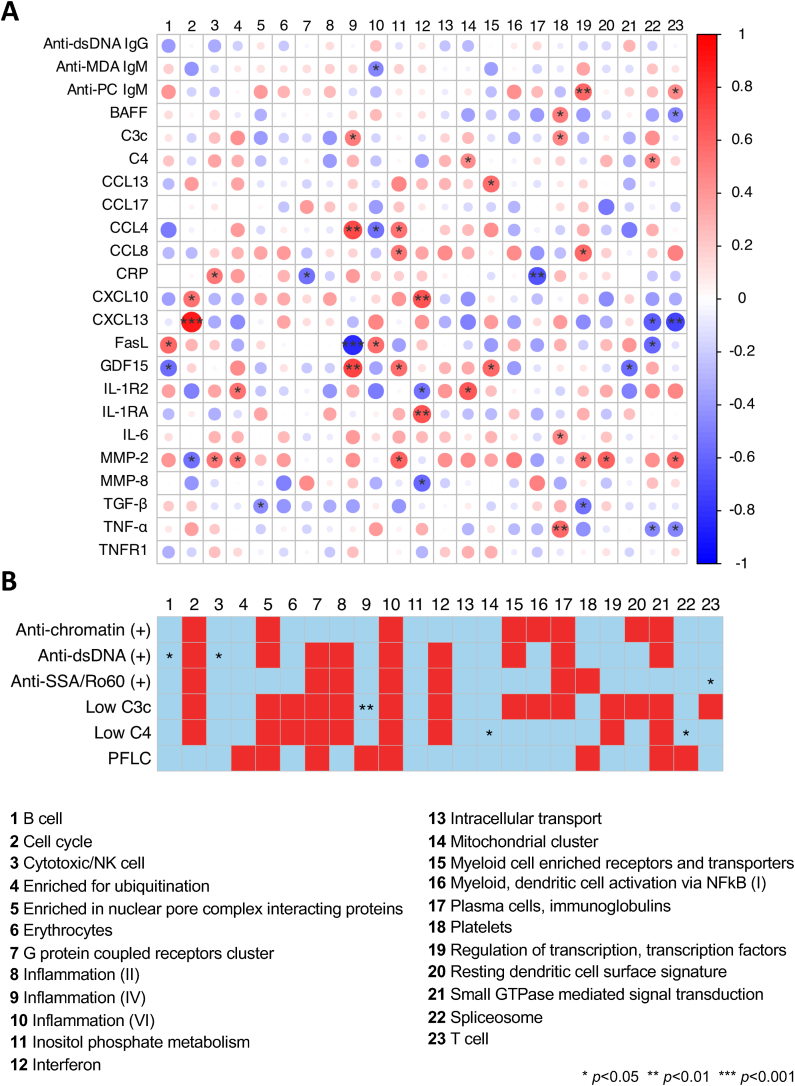
Dysregulated gene modules in relation to serological markers in patients with active CNS lupus. **(A)** The correlation heatmap shows Spearman's rank correlation coefficients for correlations between levels of selected serological markers and dysregulation scores for gene modules as measured by z-scores. **(B)** Dysregulation of gene modules in relation to autoantibody positivity or low levels of C3c or C4. The group without autoantibody positivity or low levels of C3c or C4 for each comparison was considered the reference group. Red and blue colours denote higher and lower z-scores compared with the reference group, respectively. *p*-values are derived from Mann-Whitney *U* tests. Asterisks denote statistically significant correlations or differences. Anti-dsDNA: antibodies against double-stranded DNA; anti-SSA/Ro60: antibodies against SSA/Ro60; BAFF: B cell activating factor belonging to the tumour necrosis factor family; C3c: complement component 3c; C4: complement component 4; CCL4: C-C motif chemokine ligand 4; CCL13: C-C motif chemokine ligand 13; CCL17: C-C motif chemokine ligand 17; CNS: central nervous system; CRP: C-reactive protein; CXCL13: CXC motif chemokine ligand 13; FasL: Fas ligand; GDF15: growth differentiation factor 15; Ig: immunoglobulin; IL-1R2: interleukin 1 receptor type 2; IL-1RA: interleukin 1 receptor type antagonist; IL-6: interleukin 6; MDA: malondialdehyde; MMP-2: matrix metalloproteinase 2; MMP-8: matrix metalloproteinase 8; PC: phosphorylcholine; PFLC: polyclonal free light chains of kappa and lambda type; TGF-β: transforming growth factor β; TNF-α: transforming growth factor α; TNFR1: tumour necrosis factor receptor 1.

We next assessed gene module dysregulation scores in relation to serological markers, autoantibody positivity, and low complement levels to investigate how whole-blood gene dysregulation relates to conventional immunological markers. Among patients with active CNS lupus (n = 26), dysregulation of the cell cycle gene module in whole blood correlated with serum C-X-C motif chemokine ligand 13 (CXCL13) levels (Fig. 3A; Supplementary Table S5–S27). Likewise, dysregulation of the inflammation (IV) module correlated with levels of C-C motif chemokine ligand 4 (CCL4) and growth differentiation factor 15 (GDF15), while dysregulation within the IFN module correlated with C-X-C motif chemokine ligand 10 (CXCL10) and interleukin 1 receptor antagonist (IL-1RA) levels. Dysregulation of the regulation of transcription module correlated with IgM anti-PC levels, as did dysregulation of the T cell module. Lower dysregulation scores of the B cell module and the cytotoxic/NK cell gene module were associated with anti-dsDNA positivity (Fig. 3B; Supplementary Table S28–S33).

### 3.5 Druggability analysis

**Fig. 4:**
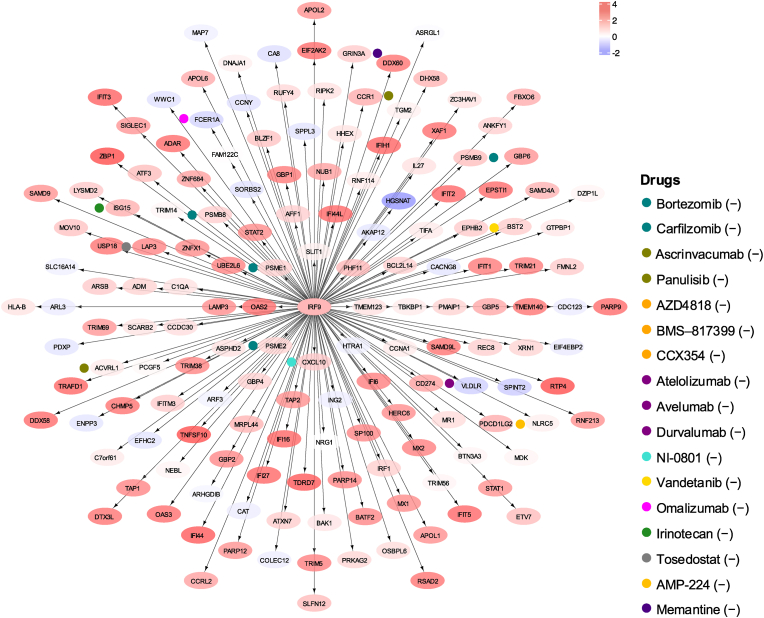
The *IRF9* signalling molecule network and annotated drug targets in patients with active CNS lupus. Genes in the interferon gene module were imputed in iRegulon through Cytoscape to generate signalling molecule networks and identify their chief regulators. One of the most enriched signalling molecule networks, based on normalised enrichment score, is plotted, with the chief regulator *IRF9* in the central node. The colour of the nodes ranges from light blue (downregulated genes) to increasing intensities of red (upregulated genes) based on the gene dysregulation (z-scores) in the CNS lupus patient subgroup 1. Coloured dots next to genes indicate drugs modulating these genes, with corresponding drug names listed to the right. Minus and plus signs denote inhibition and stimulation, respectively. Selected drugs of particular relevance are labelled, and the full list of drugs is provided in the Supplementary Material, sheet 10. CNS: central nervous system; IRF9: interferon regulatory factor 9.

The chief regulators and motifs of the most enriched signalling molecule networks, based on genes within the prominently dysregulated gene modules, are detailed in the Supplementary Table S34. The interferon regulatory factor 9 (*IRF9*) gene was identified as the main regulator in the most enriched signalling molecule network deriving from the genes in the IFN gene module, as shown for the active CNS lupus subgroup 1 in Fig. 4. Drugs associated with genes in this network included the proteasome inhibitor bortezomib and the N-methyl-D-aspartate receptor (NMDAR) antagonist memantine. Detailed results from the signalling molecule network and druggability analysis are provided in the Supplementary Material, sheet 11, and Supplementary Fig. S2–S8.

**Fig. 5:**
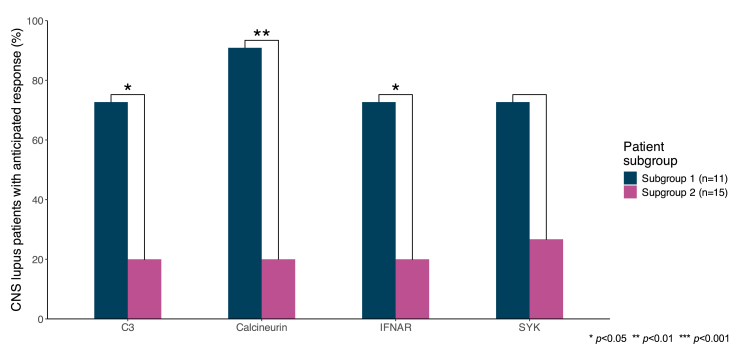
Anticipated response to inhibition of selected drug targets in patients with active CNS lupus. Bars depict proportions of patients with an anticipated benefit from inhibition of selected drug targets between active CNS lupus patient subgroups. CNS: central nervous system; IFNAR: interferon-α/β receptor; SYK: spleen tyrosine kinase.

Subsequently, we assessed anticipated response to therapies within each active CNS lupus subgroup by modulating gene targets of interest, identified as described above. Response scores from *in silico* prediction modelling are detailed in the Supplementary Table S35. A greater proportion of patients in the active CNS lupus subgroup 1 displayed an anticipated benefit from the anti-interferon-α/β receptor (IFNAR) monoclonal antibody anifrolumab (73 % versus 20.0 %; *p* = 0.015), C3 inhibitors (73 % versus 20 %; *p* = 0.015), and calcineurin inhibitors (91 % versus 20 %; *p* = 0.001) compared with subgroup 2 (Fig. 5). A numerically greater proportion of patients in subgroup 1 (72.7 %) compared with those in subgroup 2 (26.7 %) showed anticipated benefit from spleen tyrosine kinase (SYK) inhibitors, but the difference did not reach statistical significance (*p* = 0.054; Supplementary Table S36).

**Fig. 6:**
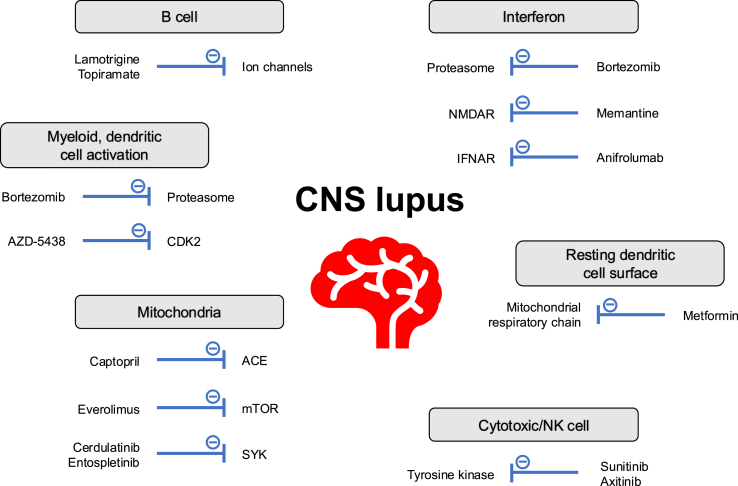
Overview of major mechanisms and effector pathways implicated in CNS lupus pathogenesis emerging as targets of future therapies. Molecular network analysis followed by druggability assessment suggested key dysregulated gene modules involving B cells, myeloid cells, dendritic cells, mitochondria, interferon, resting dendritic cell surface, and cytotoxic/NK cells. Aberrant transcriptomic signatures could be reversed by specific drugs, as schematically depicted. ACE: angiotensin-converting enzyme; CDK2: cyclin-dependent kinase 2; CNS: central nervous system; IFNAR: interferon-α/β receptor; mTOR: mammalian target of rapamycin; NMDAR: N-methyl-D-aspartate receptor; SYK: spleen tyrosine kinase; TLR: toll-like receptor 5.

An overview of results from the druggability analysis is shown in Fig. 6.

## 4 Discussion

Management of NPSLE remains a clinical challenge, largely due to marked heterogeneity and the difficulty in attributing neuropsychiatric features to SLE. These factors underscore the need for tailored treatment strategies targeting specific molecular pathways. In this study, we analysed the peripheral blood whole-genome transcriptome of SLE patients, focusing on CNS lupus. Patients with active CNS lupus exhibited distinct gene expression profiles compared to those with active SLE yet no history of NPSLE, with 1576 unique DEGs. Unsupervised clustering revealed two molecular subgroups of active CNS lupus, differing in estimated immune cell subset proportions, and suggesting divergent pathogenetic mechanisms. Specific gene modules correlated with serological markers, including the cell cycle gene module, which correlated with the B cell chemoattractant CXCL13. Molecular network analysis combined with druggability assessment and *in silico* predicted response to treatments identified potential therapeutic targets and opportunities for drug purposing, providing a basis for future clinical trials in NPSLE.

We identified mechanisms specific to patients with active CNS lupus. First, we confirmed enhanced chromosome segregation, previously reported in SLE [19]. We then demonstrated 23 dysregulated gene modules involving several immune pathways, following a pipeline previously applied in lupus nephritis (LN) [20]. These modules stratified active CNS lupus patients into two molecular subgroups. Subgroup 1 exhibited prominent downregulation of B cell, T cell, and NK cell gene modules, while subgroup 2 showed limited dysregulation. The IFN gene module was notably upregulated in subgroup 1, reinforcing the role of IFN signalling across SLE manifestations. *In silico* druggability analysis predicted a higher therapeutic response in subgroup 1 to type I IFN receptor blockade, as well as C3 and calcineurin inhibition, suggesting subgroup-specific therapeutic implications. Conversely, subgroup 2 demonstrated no strong predicted response to conventional immunosuppressants, likely reflecting greater biological heterogeneity. This subgroup may represent either misclassified NPSLE, supported by its molecular similarity to HC, or a difficult-to-treat NPSLE group, underscoring the challenge of accurate symptom attribution in clinical practice.

The molecular differences between the two subgroups prompted us to explore potential alterations in circulating immune cells. Subgroup 1 demonstrated a distinct profile, characterised by a relative abundance of myeloid cells, reflected by an elevated NLR, alongside reduced B cells, T cells, and NK cells. This may reflect increased lymphocyte infiltration into the CNS, consistent with murine models of NPSLE showing B and T cell infiltration in brain tissue [[21], [22], [23]]. However, migration is unlikely to fully explain the specific reduction in naïve and resting memory subsets, which are not typically infiltrated into inflamed tissues. One possible explanation is increased differentiation of these subsets into activated or effector cells in response to immune activation, as supported by the gene signatures observed in patients with CNS lupus. Additionally, sequestration in lymphoid organs, or alterations in lymphocyte development or survival, may also contribute to these findings. Subgroup 1 also displayed higher estimated proportions of neutrophils and plasma cells, suggesting a role for both inflammatory mechanisms and autoantibodies. In contrast, subgroup 2 showed no significant deviations from HC. Additionally, anti-dsDNA antibodies were present only in subgroup 1, with an abundance similar to what has been reported in patients with active NPSLE [24]. This finding aligns with the observed molecular differences and suggests a higher inflammatory burden in subgroup 1. Headache was more prevalent in subgroup 2, a common symptom among patients with SLE yet rarely attributed to the disease [25]. These findings imply that subgroup 2 may have been misclassified having CNS lupus and highlight the potential utility of molecular profiling to improve attribution accuracy and diagnostic precision in NPSLE.

We further explored associations between gene modules and serological markers. No strong correlations were observed with autoantibodies, except IgM anti-PC levels which correlated with the regulation of transcription and T cell gene modules. Given their inverse relationship with cardiovascular events in autoimmune diseases [26], IgM anti-PC may confer protection against thrombotic NPSLE. CXCL13 levels correlated with the cell cycle module, and CCL4 and GDF15 with an inflammation module. CXCL13 has been proposed as a potential biomarker of activity in SLE and LN [27,28], and CXCL13 and CCL4 were shown to have utility in SLE-related haemolytic anaemia, while their roles in NPSLE remain elusive [29]. GDF15 is elevated in SLE and associated with disease activity, yet, again, its role in NPSLE is not thoroughly studied [28,30]. CXCL10 correlated with the IFN module, consistent with its known induction via IFNα [31] and its association with SLE activity. Our findings suggest that CXCL10 warrants further investigation in NPSLE [28].

A prominent feature in both CNS lupus subgroups was the upregulation of IFN, particularly in subgroup 1. Druggability analysis of IFN gene-based signalling networks revealed bortezomib, carfilzomib, memantine, irinotecan, panulisib, and tosedostat as promising inhibitory agents. The proteasome inhibitors bortezomib and carfilzomib were of particular interest based on upregulation of genes within the PSMB family and, given prior reports of the clinical utility of bortezomib in refractory SLE [20,32] and NMDA receptor encephalitis [33], although adverse events limits its use [34]. Memantine, which targets the glutamatergic system through NMDA receptors [35], has been trialled for cognitive impairment in SLE without significant benefit [13]. However, participants in that trial had advanced disease, and the lack of efficacy might be due to irreversible brain damage. Irinotecan, a topoisomerase 1 inhibitor, has demonstrated efficacy in ameliorating SLE by modifying DNA relaxation and anti-dsDNA binding in two distinct mouse models of lupus [36,37]. In a case report of LN, low-dose irinotecan yielded favourable outcomes without raising safety concerns [38], though evidence in NPSLE is lacking. Targeting the phosphoinositide 3 kinase (PI3K) and mammalian target of rapamycin (mTOR) pathways with panulisib counteracts the IFN module. Panulisib was developed for treating cancer, yet the pathways it targets are also implicated in SLE [39], and our study indicates that it might offer therapeutic benefits in NPSLE. The aberrant IFN module can also be effectively reversed by the aminopeptidase inhibitor tosedostat, which has shown benefit in acute myeloid leukaemia [40].

Druggability analysis for the B cell, NK cell, and mitochondrial modules revealed several compounds that could reverse immune aberrancies among patients in the CNS lupus subgroup 1. The B cell module unveiled two noteworthy yet non-B cell-specific targets, i.e., lamotrigine and topiramate. Both drugs, classified as sodium channel blockers, are widely used in neurological and psychiatric conditions [41] and could be of interest for the symptomatic management of certain NPSLE manifestations [6]. The NK cell module uncovered potential drugs involved in the modulation of tyrosine kinases through the *ELF1* gene, including sunitinib and axitinib. Axitinib is an RTK inhibitor that enables suppression of angiogenesis via VEGF inhibition [42]. Clinical experience with axitinib is limited to cancer, but tyrosine kinases play a crucial role in transmitting signals from leukocyte antigen receptors, innate immune receptors, and cytokine receptors. This signalling is essential for the activation and infiltration of leukocytes into target organs, suggesting that axitinib could potentially reduce immune cell infiltration into the brain.

Aberrant activation of mitochondrial pathways in the CNS lupus subgroup 1 could be effectively reversed by the angiotensin-converting enzyme (ACE) inhibitor captopril and SYK inhibitors including cerdulatinib and entospletinib. A recent study that used the DNRAb+ mouse model demonstrated microglia-mediated dendritic pruning, which was reversed by depleting activated microglia. Notably, treatment with captopril, a BBB-penetrating ACE inhibitor, significantly mitigated microglia activation and improved the cognitive function of the mice [43]. In line with that study, our findings provide support for testing captopril for cognitive impairment in SLE. Activation of TLR5 pathways has been observed in SLE patients who do not attain low disease activity or remission [44,45]. In conformity, we found *TLR5* upregulation in the active CNS lupus subgroup 1. Research has identified impaired metabolism as a critical aspect of SLE pathogenesis, thereby proposing mTOR inhibitors such as everolimus as a potential therapeutic approach [46,47]. Real-world data from 27 patients with active SLE demonstrated its efficacy in treating musculoskeletal manifestations with no safety concerns; however, patients with NPSLE were not included in the study [48]. Sirolimus has been evaluated in an open-label phase 1/2 trial comprising 43 active SLE patients, yielding promising results [49]. Coupled with our results, further exploration of mTOR in NPSLE has merit. To this end, our data suggest that inhibition of SYK, which is directly linked to the B cell and the Fcγ receptors, could potentially be a therapeutic option in NPSLE [50,51].

Our study has several limitations. The cohort comprised exclusively White/Caucasian patients of European descent, limiting generalisability. The cross-sectional design precluded the examination of intra-individual transcriptomic changes over time, and the lack of cerebrospinal fluid and imaging data limited our ability to provide complementary insights. Selection bias may have occurred due to exclusion criteria such as recent use of cyclophosphamide or belimumab, cell depleting therapies, and glucocorticoids exceeding 15 mg/day of a prednisone equivalent, potentially omitting severe NPSLE flares. Nevertheless, the glucocorticoid restrictions minimised their interference with gene expression. The small sample size constituted a limitation and precluded correction for multiple comparisons in serological analyses and analyses stratified by patient subgroups. Overall, our results are hypothesis-generating and should be interpreted with caution. Among strengths of our investigation was the specific focus on active CNS lupus and the inclusion of well-defined cases of active SLE with no history of neuropsychiatric events, as a comparator group, ensuring clinically relevant comparisons. Unsupervised clustering identified distinct molecular subsets, and druggability analysis revealed therapeutic targets specific to molecular clusters and patient subgroups, highlighting the potential for precision medicine in NPSLE.

## 5 Conclusions

Gene dysregulation patterns related to innate and adaptive lymphoid immunity separated active CNS lupus patients into two distinct subgroups with differential anticipated response to type I interferon, C3, and calcineurin inhibition. Our study provides a conceptual framework for precision medicine in NPSLE and informs approaches to address the major challenge of accurately attributing neuropsychiatric features to SLE.

## CRediT authorship contribution statement

**Julius Lindblom:** Writing – review & editing, Writing – original draft, Visualization, Methodology, Investigation, Formal analysis, Data curation, Conceptualization. **Guillermo Barturen:** Writing – review & editing, Supervision, Methodology, Investigation, Formal analysis, Data curation, Conceptualization. **Lorenzo Beretta:** Writing – review & editing, Supervision, Methodology, Investigation, Formal analysis, Data curation. **Daniel Toro-Domínguez:** Writing – review & editing, Supervision, Methodology, Investigation, Formal analysis. **Elena Carnero-Montoro:** Writing – review & editing, Supervision, Methodology, Investigation, Formal analysis. **Maria Orietta Borghi:** Writing – review & editing, Supervision, Methodology, Investigation, Data curation. **Jessica Castillo:** Writing – review & editing, Supervision, Methodology, Investigation, Formal analysis. **Ellen Iacobaeus:** Writing – review & editing, Supervision, Methodology, Investigation, Formal analysis. **Yvonne Enman:** Writing – review & editing, Supervision, Methodology, Investigation, Formal analysis. **Chandra Mohan:** Writing – review & editing, Supervision, Methodology, Investigation, Formal analysis. **Marta E. Alarcón-Riquelme:** Writing – review & editing, Supervision, Methodology, Investigation, Funding acquisition, Formal analysis, Data curation. **Dionysis Nikolopoulos:** Writing – review & editing, Writing – original draft, Visualization, Supervision, Methodology, Investigation, Funding acquisition, Formal analysis, Conceptualization. **Ioannis Parodis:** Writing – review & editing, Writing – original draft, Visualization, Supervision, Software, Methodology, Investigation, Funding acquisition, Formal analysis, Data curation, Conceptualization.

## Ethical approval

This study involves human participants. It was reviewed, and approved by local ethics committees at all recruiting centres: Comitato Etico Area 2, Fondazione IRCCS Ca Granda Ospedale Maggiore Policlinico di Milano and University of Milan (approval no. 425 bis 19 November 19 2014 and no. 671_2018 19 September 19 2018); Klinikum der Universitaet zu Koeln, Cologne, Germany; Comite d Ethique Hospitalo-Facultaire, Pole de pathologies rhumatismales systemiques et inflammatoires, Institut de Recherche Experimentale et Clinique, Universite catholique de Louvain, Brussels, Belgium; Csongrad Megyei Kormanyhivatal, University of Szeged, Szeged, Hungary; Comite Etica de Investigacion Clinica del Hospital Clínic de Barcelona, Hospital Clinic I Provicia, Institut d Investigacions Biomediques August Pi i Sunyer, Barcelona, Spain; Comite de Etica e la Investigacion de Centro de Granada (CEI—Granada), Servicio Andaluz de Salud, Hospital Universitario Reina Sofía Cordoba, Spain; Comissao de ética para a Saude—CES do CHP, Centro Hospitalar do Porto, Portugal; Comite de Protection des Personnes Ouest VI, Centre Hospitalier Universitaire de Brest, Hospital de la Cavale Blanche, Avenue Tanguy Prigent 29609, Brest, France; DEAS—Commission Cantonale d ethique de la recherche Hopitaux universitaires de Geneve, Hospitaux Universitaires de Genève, Switzerland; Andalusian Public Health System Biobank, Granada, Spain; Commissie Medische Ethiek UZ KU Leuven/Onderzoek, Katholieke Universiteit Leuven, Belgium; Ethikkommission, Charite, Berlin, Germany; Ethikkommission, Medizinische Hochschule Hannover, Germany. Participants gave informed consent to participate in the study before taking part. The study protocol for the present analysis was reviewed and approved by the Swedish Ethical Review Authority (approval no. 2022-03907-01).

## Disclaimer

The content of this publication reflects only the authors’ view and the JU is not responsible for any use that may be made of the information it contains.

## Funding

IP has received grants from the 10.13039/501100007949Swedish Rheumatism Association (R-995882), King Gustaf V's 80-year Foundation (FAI-2023-1055), 10.13039/501100007687Swedish Society of Medicine (SLS-974449), Nyckelfonden (OLL-1000881), Professor Nanna Svartz Foundation (2021-00436), Ulla and Roland Gustafsson Foundation (2024-43), Region Stockholm (FoUI-1004114), and 10.13039/501100004047Karolinska Institutet. DN has received grants from the 10.13039/501100007949Swedish Rheumatism Association (R-995557), King Gustaf V's 80-year Foundation (FAI-2023-1006), Ulla and Roland Gustafsson Foundation (2024-49), 10.13039/501100009806Ulla and Gustafaf Uggla Foundation (2023–025029), and 10.13039/501100004047Karolinska Institutet. We acknowledge scientific support from the Exploring Inflammation in Health and Disease (X-HiDE) Consortium, a research profile at Örebro University funded by the 10.13039/100003077Knowledge Foundation (grant no. 20200017). This work was supported by 10.13039/501100010767Innovative Medicines Initiative (10.13039/501100010767IMI) Joint Undertaking (JU) for the PRECISESADS project under the grant agreement no. 115565. It also received funding from the IMI 2 JU for the Taxonomy, Treatment, Targets and Remission (3 TR) project under the grant agreement no. 831434. The JU receives support from the European Union's Horizon 2020 research and innovation programme and 10.13039/100013322European Federation of Pharmaceutical Industries and Associations (10.13039/100013322EFPIA).

## Declaration of competing interest

The authors declare the following financial interests/personal relationships which may be considered as potential competing interests: Ioannis Parodis reports a relationship with Amgen Inc that includes: consulting or advisory and funding grants. Ioannis Parodis reports a relationship with AstraZeneca that includes: consulting or advisory and funding grants. Ioannis Parodis reports a relationship with Aurinia Pharmaceuticals Inc that includes: consulting or advisory and funding grants. Ioannis Parodis reports a relationship with Bristol Myers Squibb Co that includes: consulting or advisory and funding grants. Ioannis Parodis reports a relationship with Eli Lilly and Company that includes: consulting or advisory and funding grants. Ioannis Parodis reports a relationship with Gilead Sciences Inc that includes: consulting or advisory and funding grants. Ioannis Parodis reports a relationship with GSK that includes: consulting or advisory and funding grants. Ioannis Parodis reports a relationship with Janssen Pharmaceuticals Inc that includes: consulting or advisory and funding grants. Ioannis Parodis reports a relationship with Novartis that includes: consulting or advisory and funding grants. Ioannis Parodis reports a relationship with Otsuka Pharmaceutical Co Ltd that includes: consulting or advisory and funding grants. Ioannis Parodis reports a relationship with Roche that includes: consulting or advisory and funding grants. If there are other authors, they declare that they have no known competing financial interests or personal relationships that could have appeared to influence the work reported in this paper.

## Data Availability

Data are available upon reasonable request. Raw data remain the property of the PRECISESADS consortium and are protected under the European General Data Protection Regulation (GDPR). Metadata and aggregated processed data are available upon reasonable request from the corresponding author and from the European Genome-Phenome Archive (EGA).
