# Supplementary material for: Dysregulation of innate and adaptive lymphoid immunity may have implications for symptom attribution and predict responses to targeted therapies in neuropsychiatric systemic lupus erythematosus
Source: J Transl Autoimmun. 2025 Jun 11;11:100296. doi: 10.1016/j.jtauto.2025.100296 (PMC12266536; doi:10.1016/j.jtauto.2025.100296)
Supplement: Multimedia component 1 [file mmc1.pdf]

Supplementary Material

Table of contents

Supplementary Table S1. Main inclusion and exclusion criteria 4

Physician-reported central nervous system involvement according to the PRECISESADS case report form (CRF) 5

List of local investigators from the participating clinical sites 6

Supplementary Figure S1. Dysregulation scores of gene modules in patients with active CNS lupus 10

Supplementary Table S2. Z-scores of gene modules in patient subgroups with active CNS lupus 11

Supplementary Table S3. Estimated relative immune cell type proportions across CNS lupus subgroups, patients with active non-neuropsychiatric-SLE, and healthy controls 12

Supplementary Table S4. Estimated NLR across CNS lupus subgroups, patients with active non-neuropsychiatric-SLE, and healthy controls 13

Supplementary Table S5. Correlations between serological markers and z-scores of the "B cell" gene module in patients with active CNS lupus 14

Supplementary Table S6. Correlations between serological markers and z-scores of the "cell cycle" gene module in patients with active CNS lupus 15

Supplementary Table S7. Correlations between serological markers and z-scores of the "cytotoxic/NK cell" gene module in patients with active CNS lupus 16

Supplementary Table S8. Correlations between serological markers and z-scores of the "enriched for ubiquitination" gene module in patients with active CNS lupus 17

Supplementary Table S9. Correlations between serological markers and z-scores of the "enriched in nuclear pore complex interacting proteins" gene module in patients with active CNS lupus 18

Supplementary Table S10. Correlations between serological markers and z-scores of the "erythrocytes" gene module in patients with active CNS lupus 19

Supplementary Table S11. Correlations between serological markers and z-scores of the "G protein coupled receptors cluster" gene module in patients with active CNS lupus 20

Supplementary Table S12. Correlations between serological markers and z-scores of the "inflammation (II)" gene module in patients with active CNS lupus 21

Supplementary Table S13. Correlations between serological markers and z-scores of the "inflammation (IV)" gene module in patients with active CNS lupus 22

Supplementary Table S14. Correlations between serological markers and z-scores of the "inflammation (VI)" gene module in patients with active CNS lupus 23

Supplementary Table S15. Correlations between serological markers and z-scores of the "inositol phosphate metabolism" gene module in patients with active CNS lupus 24

Supplementary Table S16. Correlations between serological markers and z-scores of the "interferon" gene module in patients with active CNS lupus 25

Supplementary Table S17. Correlations between serological markers and z-scores of the "intracellular transport" gene module in patients with active CNS lupus 26

Supplementary Table S18. Correlations between serological markers and z-scores of the "mitochondrial cluster" gene module in patients with active CNS lupus.................................27

Supplementary Table S19. Correlations between serological markers and z-scores of the "myeloid cell enriched receptors and transporters" gene module in patients with active CNS lupus 28

Supplementary Table S20. Correlations between serological markers and z-scores of the "Myeloid, dendritic cell activation via NFkB (I)" gene module in patients with active CNS lupus 29

Supplementary Table S21. Correlations between serological markers and z-scores of the "plasma cells, immunoglobulins" gene module in patients with active CNS lupus 30

Supplementary Table S22. Correlations between serological markers and z-scores of the "platelets" gene module in patients with active CNS lupus..31

Supplementary Table S23. Correlations between serological markers and z-scores of the "regulation of transcription, transcription factors" gene module in patients with active CNS lupus 32

Supplementary Table S24. Correlations between serological markers and z-scores of the "resting dendritic cell surface signature" gene module in patients with active CNS lupus.......33

Supplementary Table S25. Correlations between serological markers and z-scores of the "small GTPase mediated signal transduction" gene module in patients with active CNS lupus 34

Supplementary Table S26. Correlations between serological markers and z-scores of the "spliceosome" gene module in patients with active CNS lupus 35

Supplementary Table S27. Correlations between serological markers and z-scores of the "T cell" gene module in patients with active CNS lupus 36

Supplementary Table S28. Z-scores of gene modules in patients with active CNS lupus and anti-chromatin positivity versus negative patients 37

Supplementary Table S29. Z-scores of gene modules in patients with active CNS lupus and anti-dsDNA positivity versus negative patients...38

Supplementary Table S30. Z-scores of gene modules in patients with active CNS lupus and anti-SSA/Ro60 positivity versus negative patients 39

Supplementary Table S31. Z-scores of gene modules in patients with active CNS lupus and low versus patients with normal/high levels of C3c 40

Supplementary Table S32. Z-scores of gene modules in patients with active CNS lupus and low versus patients with normal/high levels of C4 41

Supplementary Table S33. Z-scores of gene modules in patients with active CNS lupus and PFLC versus patients with no PFLC 42
Supplementary Table S34. The most enriched signalling molecule networks in patients with active CNS lupus 43
Supplementary Figure S2. The *TBX2* signalling molecule network and annotated drug targets in patients with active CNS lupus 44
Supplementary Figure S3. The *ELF1* signalling molecule network and annotated drug targets in patients with active CNS lupus 45
Supplementary Figure S4. The *CEBPE* signalling molecule network and annotated drug targets in patients with active CNS lupus 46
Supplementary Figure S5. The *ZBTB33* signalling molecule network and annotated drug targets in patients with active CNS lupus 47
Supplementary Figure S6. The *SP1* signalling molecule network and annotated drug targets in patients with active CNS lupus 48
Supplementary Figure S7. The *ZNF143* signalling molecule network and annotated drug targets in patients with active CNS lupus 49
Supplementary Figure S8. The *ZNF143* signalling molecule network and annotated drug targets in patients with active CNS lupus 50
Supplementary Table S35. Response scores to selected targets in patients with active CNS lupus 51
Supplementary Table S36. CNS lupus patients with anticipated response to selected drug targets 52

Supplementary Table S1. Main inclusion and exclusion criteria

Inclusion criteria	Exclusion criteria
**For all patients**	**For all patients**
- Age ≥18 years<br>- Diagnosed according to the prevailing criteria for systemic lupus erythematosus (SLE)<br>- Informed consent signed	- Paediatric lupus<br>- Drug-induced lupus<br>- Severe nephrotic syndrome with proteinuria ≥3.5 g/day<br>- Patients with stable doses of prednisone equivalent >15 mg/day for the last 3 months or with IV corticosteroids in the last 3 months<br>- Patients under immunosuppressant treatment in the last 3 months prior to recruitment and patients with combined therapy using two or more immunosuppressants:<br>• Methotrexate ≥25mg/week<br>• Azathioprine ≥2.5mg/kg/day<br>• Cyclosporine A >3mg/kg/day<br>• Mycophenolate mofetil >2g/day<br>- Treatment with cyclophosphamide (any dose or route of administration) or belimumab in the past 6 months<br>- Patients on depleting therapy such as rituximab in the last year<br>- Chronic HBV or HCV infection<br>- Patients who are also diagnosed according to the prevailing criteria for one of the following autoimmune diseases:<br>• Rheumatoid arthritis (RA)<br>• Scleroderma or systemic sclerosis (SSc)<br>• Primary Sjögren's syndrome (pSjS)<br>• Primary antiphospholipid syndrome (pAPS)<br>• Mixed connective tissue disease (MCTD)<br>• Patients with undifferentiated connective tissue disease (UCTD) for over 1 year and that do not fulfill the diagnosis of any of the above diseases or SLE
	**For controls**
	- Individuals on chronic medication<br>- Individuals suffering from any inflammatory autoimmune, allergic or infectious condition, and with a history of autoimmune disease, particularly thyroid disease or other diseases that may modify cellular profiles in blood

Adapted from "Integrative Analysis Reveals a Molecular Stratification of Systemic Autoimmune Diseases." Arthritis Rheum. 2021;73(6):1073-1085. Copyright 2021 by the American College of Rheumatology. Adapted with permission. IV: intravenous, HBV: hepatitis B virus, HCV: hepatitis C virus.

Physician-reported central nervous system involvement according to the PRECISESADS case report form (CRF)

Presence of any of the following conditions, in the absence of infections, metabolic or endocrine disturbances, adverse drug reactions, or any other predisposing condition unrelated to systemic autoimmune diseases:

- Transitory ischaemic attacks
- Ischaemic stroke
- Mild-to-moderate cognitive disfunction with impaired attention or visual memory or verbal memory, or impaired executive function and psychomotor speed
- Seizures
- Chorea
- Acute confusional state characterised by acute onset, fluctuating level of consciousness with decreased attention
- Psychiatric disorders characterised by delusions (false beliefs refuted by objective evidence) or hallucinations (perceptions in the absence of external stimuli)
- Transverse myelitis
- Aseptic meningitis
- Inflammatory optic neuritis
- Ischaemic or thrombotic optic neuropathy

Reported as “Past” if observed and resolved, “Present” if observed at the visit, “No” if never observed or reported, or “Unk” if not verified.

List of local investigators from the participating clinical sites

Lorenzo Beretta[1], Barbara Vigone[1], Jacques-Olivier Pers[2], Alain Saraux[2], Valérie Devauchelle-Pensec[2], Divi Cornec[2], Sandrine Jousse-Joulin[2], Bernard Lauwerys[3], Julie Ducreux[3], Anne-Lise Maudoux[3], Carlos Vasconcelos[4], Ana Tavares[4], Esmeralda Neves[4], Raquel Faria[4], Mariana Brandão[4], Ana Campar[4], António Marinho[4], Fátima Farinha[4], Isabel Almeida[4], Miguel Angel Gonzalez-Gay Mantecón[5], Ricardo Blanco Alonso[5], Alfonso Corrales Martínez[5], Ricard Cervera[6], Ignasi Rodríguez-Pintó[6], Gerard Espinosa[6], Rik Lories[7], Ellen De Langhe[7], Nicolas Hunzelmann[8], Doreen Belz[8], Torsten Witte[9], Niklas Baerlecken[9], Georg Stummvoll[10], Michael Zauner[10], Michaela Lehner[10], Eduardo Collantes[11], Rafaela Ortega-Castro[11], Mª Angeles Aguirre-Zamorano[11], Alejandro Escudero-Contreras[11], Mª Carmen Castro-Villegas[11], Norberto Ortego[12], María Concepción Fernández Roldán[12], Enrique Raya[13], Inmaculada Jiménez Moleón[13], Enrique de Ramon[14], Isabel Díaz Quintero[14], Pier Luigi Meroni[15], Maria Gerosa[15], Tommaso Schioppo[15], Carolina Artusi[15], Carlo Chizzolini[16], Aleksandra Zuber[16], Donatienne Wynar[16], Laszló Kovács[17], Attila Balog[17], Magdolna Deák[17], Márta Bocskai[17], Sonja Dulic[17], Gabriella Kádár[17], Falk Hiepe[18], Velia Gerl[18], Silvia Thiel[18], Manuel Rodriguez Maresca[19], Antonio López-Berrio[19], Rocío Aguilar-Quesada[19], Héctor Navarro-Linares[19]

Affiliations

1. Referral Center for Systemic Autoimmune Diseases, Fondazione IRCCS Ca' Granda Ospedale Maggiore Policlinico di Milano, Italy

2. Centre Hospitalier Universitaire de Brest, Hospital de la Cavale Blanche, Brest, France

3. Pôle de pathologies rhumatismales systémiques et inflammatoires, Institut de Recherche Expérimentale et Clinique, Université catholique de Louvain, Brussels, Belgium

4. Centro Hospitalar do Porto, Portugal

5. Servicio Cantabro de Salud, Hospital Universitario Marqués de Valdecilla, Santander, Spain

6. Hospital Clinic I Provicia, Institut d'Investigacions Biomèdiques August Pi i Sunyer, Barcelona, Spain

7. Katholieke Universiteit Leuven, Belgium

8. Klinikum der Universitaet zu Koeln, Cologne, Germany

9. Medizinische Hochschule Hannover, Germany

10. Medical University Vienna, Vienna, Austria

11. Servicio Andaluz de Salud, Hospital Universitario Reina Sofía Córdoba, Spain

12. Servicio Andaluz de Salud, Complejo hospitalario Universitario de Granada (Hospital Universitario San Cecilio), Spain

13. Servicio Andaluz de Salud, Complejo hospitalario Universitario de Granada (Hospital Virgen de las Nieves), Spain

14. Servicio Andaluz de Salud, Hospital Regional Universitario de Málaga, Spain

15. Università degli studi di Milano, Milan, Italy

16. Hospitaux Universitaires de Genève, Switzerland

17. University of Szeged, Szeged, Hungary

18. Charite, Berlin, Germany

19. Andalusian Public Health System Biobank, Granada, Spain

Statistical and bioinformatic analysis

Genes with low counts were removed, and the raw counts of the remaining genes were transformed into $\log_2$ counts per million (CPM). For duplicated gene symbols, the mean count was used. To identify pathways specifically enriched in active CNS lupus, we performed differential gene expression analysis using the limma R package [1], incorporating voom transformation and adjusting for age, sex, sequencing batch, and RNA integrity number (RIN). We first compared patients with active CNS lupus (n=26) with HC (n=497). Next, we conducted a separate differential gene expression analysis comparing patients with active SLE but no history of NPSLE (n=38) with HC (n=497). Significant differentially expressed genes (DEGs) from both analyses were visualised using the eulerr R package [2]. Pathway enrichment analysis was performed by over-representation analysis (ORA) using the ClusterProfiler [3] and ReactomePA [4] R packages. This analysis focused on genes specific for active CNS to identify enriched Gene Ontology (GO) terms [5], as well as Reactome [6] and Kyoto Encyclopedia of Genes and Genomes (KEGG) pathways [7].

We identified gene modules in the active CNS group using weighted gene co-expression network analysis (WGCNA) based on full transcriptome data and we functionally annotated all identified gene modules based on gene expression data from Chaussabel et al. [8] and Li et al. [9] For each patient with active CNS lupus, we calculated mean z-scores compared to age- and sex-matched HC at a 1:5 ratio (n=130) to assess dysregulation, as shown in the formula below, adopting a similar methodology as that recently reported by Toro-Domínguez et al [10].

$$z_i = \mathrm{mean}\left(\frac{Gene_{CNS\,lupus\,patient} - mean(Gene_{HC})}{sd(Gene_{HC})}\right)_{\underset{1\to n}{\mathrm{Gene}}}$$

In this formula, $Gene_{CNS\ lupus\ patient}$ denotes the expression of a gene in an individual patient with active CNS lupus, $Gene_{HC}$ denotes the expression of the same gene in all age- and sex-matched HC, and n indicates the number of genes from the module i.

Dysregulated gene modules were clustered by their dysregulation scores using hierarchical clustering with the Ward method to yield gene module clusters and patient subgroups, and the pheatmap R package was employed for visualisation [11].

CIBERSORTx [12] was used with the CPM gene expression values as input to estimate the relative proportions of 22 immune cell types, using the LM22 signature matrix (naïve B cells, memory B cells, plasma cells, CD8+ T cells, naïve CD4+ T cells, resting memory CD4+ T cells, activated memory CD4+ T cells, follicular helper T cells, regulatory T cells, gamma delta (γδ) T cells, resting natural killer [NK] cells, activated NK cells, monocytes, M0 macrophages, M1 macrophages, M2 macrophages, resting dendritic cells, activated dendritic cells, resting mast cells, activated mast cells, eosinophils, and neutrophils).

Correlation analyses were performed between dysregulation scores of gene modules and serum levels of selected serological markers using Spearman's rank correlation coefficients, while the Mann-Whitney *U* test was used to assess gene dysregulation in relation to positivity for conventional autoantibodies, based on cut-offs as recommended by the assay manufacturer [13].

Gene modules with a mean |z-score| >0.5 in at least one patient subgroup were analysed using iRegulon [14] through Cytoscape [15] to generate signalling molecule networks and identify their primary regulators i.e., key transcription factors and their direct transcriptional targets, through a genome-wide ranking-and-recovery approach based on systems biology. A normalised enrichment score (NES) ≥3.0 was considered high, as previously suggested [14]. Genes within the most enriched signalling molecule network were next assessed for druggability using the Drug Gene Interaction

database (DGIdb) via the rDGIdb R package [16]. Upregulated and downregulated genes in each patient subgroup with a mean |z-score| >0.5 in the respective gene module were annotated along with their corresponding inhibitors or stimulators identified in the most enriched signalling molecule network.

Gene expression data were also analysed for druggability using the hipathia R package [17], as per its authors' instructions. The gene counts of patients with active CNS lupus were processed using trimmed mean of M-values (TMM) normalisation, log_2 conversion, and normalisation against those of matched HC (n=130). Response scores for modulating selected targets were calculated for each patient as the absolute change in gene expression before and after target inhibition, simulating inhibition with a multiplication factor 0.1. The targets tested were chosen based on results from the preceding differential gene expression and druggability analyses, and some additional were added to the list of input targets based on expert opinion. For each individual patient, favourable anticipated response to a drug was defined as a response score equal to or greater than the mean response score of all patients.

Comparisons of unrelated continuous data were made using the Mann-Whitney *U* test, associations between unrelated binomial variables were investigated using Pearson's chi squared (χ^2) or Fisher's exact tests, and correlations were assessed using Pearson or Spearman's rank correlation coefficients, as appropriate. All p-values <0.05 were considered statistically significant, and a false discovery rate (FDR)-corrected p-value <0.05 (Benjamini-Hochberg) was considered statistically significant for the differential gene expression analysis. Analyses were performed using the R software version 4.4.1 (R Foundation for Statistical Computing, Vienna, Austria).

Supplementary Figure S1. Dysregulation scores of gene modules in patients with active CNS lupus

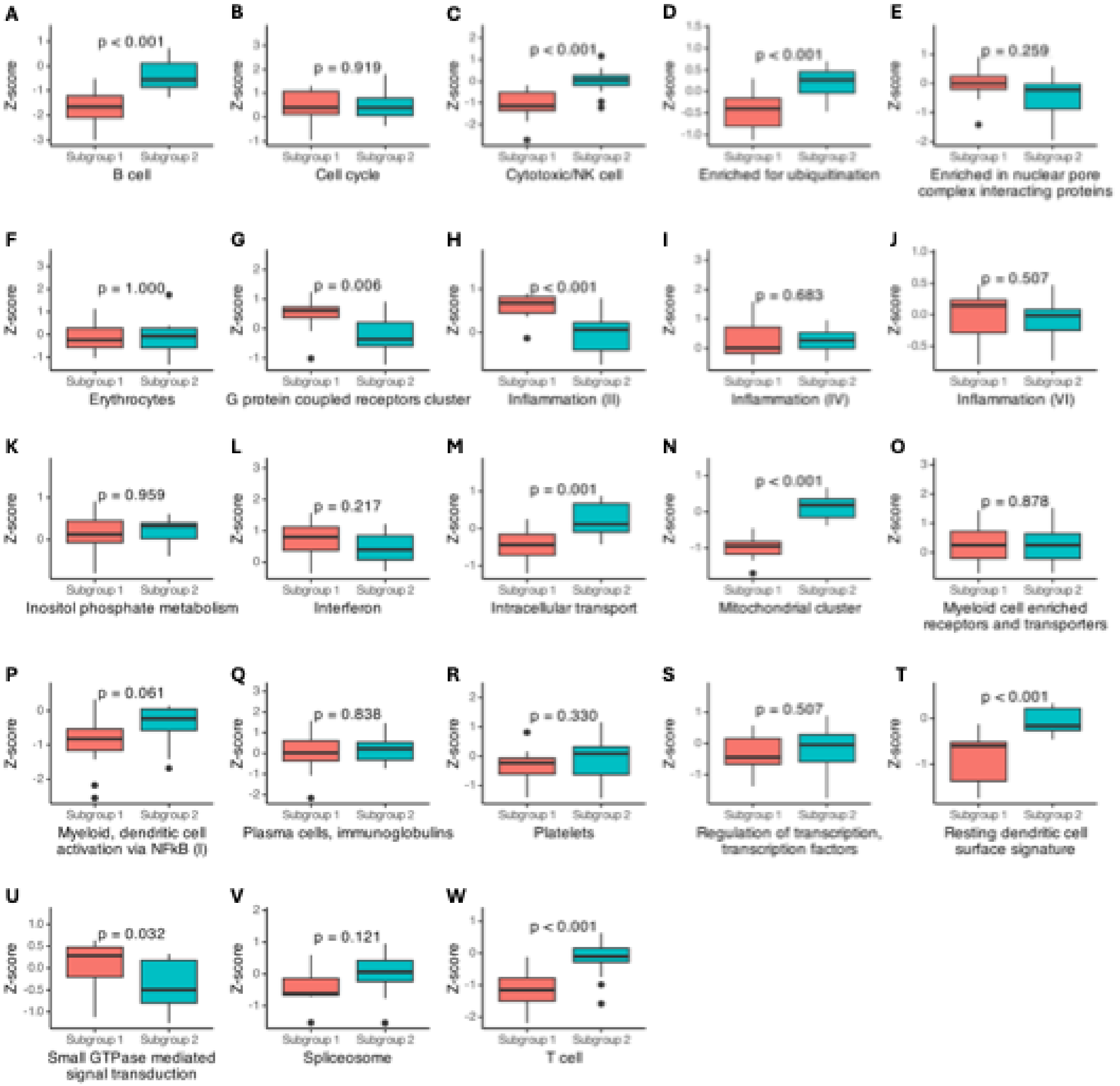

The box plots show the median dysregulation scores and interquartile ranges of gene modules, as measured by z-scores, between active CNS lupus patient subgroups. P-values were derived from Mann-Whitney *U* tests.

CNS: central nervous system.

Supplementary Table S2. Z-scores of gene modules in patient subgroups with active CNS lupus

	Active CNS lupus subgroup 1	Active CNS lupus subgroup 2	*p*-value
	**n=11**	**n=15**	
**B cell**	-1.66 (-2.10–-1.22)	-0.55 (-0.87– 0.12)	**<0.001**
**Cell cycle**	0.39 (0.12–1.07)	0.39 (0.07–0.79)	0.919
**Cytotoxic/NK cell**	-1.14 (-1.37–-0.53)	0.08 (-0.21–0.28)	**<0.001**
**Enriched for ubiquitination**	-0.40 (-0.80–-0.16)	0.27 (-0.04–0.46)	**<0.001**
**Enriched in nuclear pore complex interacting proteins**	0.00 (-0.20–0.26)	-0.23 (-0.88–-0.01)	0.259
**Erythrocytes**	-0.24 (-0.56–0.28)	-0.07 (-0.58–0.27)	1.000
**G protein coupled receptors cluster**	0.61 (0.36–0.74)	-0.36 (-0.59–0.20)	**0.006**
**Inflammation II**	0.68 (0.45–0.82)	0.07 (-0.40–0.23)	**<0.001**
**Inflammation IV**	0.01 (-0.17–0.71)	0.27 (0.00–0.53)	0.683
**Inflammation VI**	0.15 (-0.29–0.24)	-0.02 (-0.25–0.09)	0.507
**Inositol phosphate metabolism**	0.12 (-0.07–0.45)	0.33 (0.02–0.40)	0.959
**Interferon**	0.80 (0.37–1.12)	0.39 (0.08–0.85)	0.217
**Intracellular transport**	-0.44 (-0.72–-0.16)	0.12 (-0.09–0.67)	**0.001**
**Mitochondrial cluster**	-0.95 (-1.17–-0.83)	0.19 (-0.15–0.36)	**<0.001**
**Myeloid cell enriched receptors and transporters**	0.25 (-0.20–0.72)	0.25 (-0.21–0.64)	0.878
**Myeloid, dendritic cell activation via NFkB (I)**	-0.82 (-1.16–-0.53)	-0.23 (-0.57–0.05)	0.061
**Plasma cells, immunoglobulins**	0.02 (-0.35–0.61)	0.22 (-0.31–0.53)	0.838
**Platelets**	-0.23 (-0.61–-0.07)	0.09 (-0.62–0.32)	0.330
**Regulation of transcription, transcription factors**	-0.45 (-0.67–0.16)	-0.05 (-0.59–0.28)	0.507
**Resting dendritic cell surface signature**	-0.60 (-1.37–-0.52)	-0.16 (-0.26–0.21)	**<0.001**
**Small GTPase mediated signal transduction**	0.29 (-0.20–0.47)	-0.49 (-0.80–0.18)	**0.032**
**Spliceosome**	-0.60 (-0.67–-0.14)	0.06 (-0.23–0.43)	0.121
**T cell**	-1.15 (-1.51–-0.78)	-0.10 (-0.28–0.15)	**<0.001**

Data are presented as median (interquartile range). The total number of patients with available data is indicated. All *p*-values are derived from non-parametrical Mann-Whitney *U* tests. Statistically significant *p* values are in bold.

CNS: central nervous system; NK: natural killer.

Supplementary Table S3. Estimated relative immune cell type proportions across CNS lupus subgroups, patients with active non-neuropsychiatric-SLE, and healthy controls

	Active CNS lupus subgroup 1<br>n=11	Active CNS lupus subgroup 2<br>n=15	Active non-neuropsychiatric SLE<br>n=38	HC<br>n=130
**Naive B cells**	0.57	2.78	2.62	3.51
**Memory B cells**	1.08	0.51	0.82	1.05
**Plasma cells**	0.71	0.42	0.50	0.41
**CD8+ T cells**	5.70	12.23	7.72	9.28
**Naive CD4+ T cells**	3.01	5.03	5.47	7.13
**Resting memory CD4+ T cells**	8.89	14.89	11.67	15.76
**Activated memory CD4+ T cells**	1.34	0.79	0.79	0.57
**Follicular helper T cells**	0.00	0.00	0.00	0.00
**Regulatory T cells**	0.02	0.02	0.19	0.09
**Gamma delta T cells**	0.00	0.00	0.00	0.01
**Resting NK cells**	4.66	8.41	7.07	9.11
**Activated NK cells**	0.43	0.37	0.22	0.39
**Monocytes**	15.16	17.90	16.60	14.56
**M0 macrophages**	0.27	0.10	0.27	0.19
**M1 macrophages**	0.01	0.00	0.00	0.00
**M2 macrophages**	0.29	0.43	0.34	0.55
**Resting dendritic cells**	0.00	0.00	0.00	0.00
**Activated dendritic cells**	0.43	0.21	0.29	0.06
**Resting mast cells**	1.87	1.72	1.48	1.45
**Activated mast cells**	0.00	0.00	0.00	0.00
**Eosinophils**	0.00	0.00	0.00	0.01
**Neutrophils**	55.56	34.19	43.92	35.87
**Total sum**	100.0	100.0	99.97	100.00

Data are presented as percentage.

CNS: central nervous system; NK: natural killer; NP: neuropsychiatric; SLE: systemic lupus erythematosus.

Supplementary Table S4. Estimated NLR across CNS lupus subgroups, patients with active non-neuropsychiatric-SLE, and healthy controls

	**Active CNS lupus subgroup 1**	**Active CNS lupus subgroup 2**	**Active non-neuropsychiatric SLE**	**HC**
	**n=11**	**n=15**	**n=38**	**n=130**
**NLR; mean (s.d.)**	2.19 (0.64)	0.78 (0.23)	1.45 (1.01)	0.82 (0.40)
**Active CNS lupus subgroup 1 vs subgroup 2 *p*-value**	**Active CNS lupus subgroup 1 vs non-neuropsychiatric SLE *p*-value**	**Active CNS lupus subgroup 2 vs non-neuropsychiatric SLE *p*-value**	**Active CNS lupus subgroup 1 vs HC *p*-value**	**Active CNS lupus subgroup 2 vs HC *p*-value**
**<0.001**	**0.004**	**0.010**	**<0.001**	0.726

Data are presented as the mean ± standard deviation. All *p*-values are derived from non-parametrical Mann-Whitney *U* tests. Statistically significant p-values are in bold.

CNS: central nervous system; HC: healthy controls; NLR: neutrophil to lymphocyte ratio; s.d.: standard deviation; SLE: systemic lupus erythematosus.

Supplementary Table S5. Correlations between serological markers and z-scores of the “B cell” gene module in patients with active CNS lupus

Serological marker	n	Coefficient	*p*-value
**Anti-dsDNA IgG**	21	-0.38	0.088
**Anti-MDA IgM**	21	0.19	0.397
**Anti-PC IgM**	21	0.41	0.064
**BAFF**	20	0.14	0.569
**C3c**	21	0.11	0.650
**C4**	21	0.23	0.313
**CCL4**	14	-0.51	0.062
**CCL8**	14	-0.28	0.334
**CCL13**	14	-0.26	0.366
**CCL17**	14	0.00	0.994
**CRP**	15	0.00	0.990
**CXCL10**	14	-0.36	0.203
**CXCL13**	14	-0.10	0.737
**FasL**	14	0.59	**0.027**
**GDF15**	14	-0.57	**0.034**
**IL-1R2**	14	0.36	0.203
**IL-1RA**	14	-0.26	0.366
**IL-6**	20	0.12	0.613
**MMP-2**	15	0.41	0.132
**MMP-8**	14	-0.01	0.970
**TGF-ß**	20	0.21	0.380
**TNF-α**	20	0.09	0.719
**TNFR1**	14	-0.31	0.281

Spearman's rank correlation coefficients of correlations between levels of different serological markers and Z-scores of the B cell gene module. The total number of patients with available data is indicated. Statistically significant *p*-values are in bold. Only comparisons with sufficient numbers of observations (n≥10) are included.

anti-dsDNA: antibodies against double-stranded DNA; BAFF: B cell activating factor belonging to the tumour necrosis factor family; C3c: complement component 3c; C4: complement component 4; CCL4: C-C motif chemokine ligand 4; CCL8: C-C motif chemokine ligand 8; CCL13: C-C motif chemokine ligand 13; CCL17: C-C motif chemokine ligand 17; CNS: central nervous system; CRP: C-reactive protein; CXCL10: CXC motif chemokine ligand 10; CXCL13: CXC motif chemokine ligand 13; FasL: Fas ligand; GDF15: growth differentiation factor 15; Ig: immunoglobulin; IL-1R2: interleukin 1 receptor type 2; IL-1RA: interleukin 1 receptor type antagonist; IL-6: interleukin 6; MDA: malondialdehyde; MMP-2: matrix metalloproteinase 2; MMP-8: matrix metalloproteinase 8; PC: phosphorylcholine; TGF-β: transforming growth factor β; TNF-α: transforming growth factor α; TNFR1: tumour necrosis factor receptor 1.

Supplementary Table S6. Correlations between serological markers and z-scores of the "cell cycle" gene module in patients with active CNS lupus

Serological marker	n	Coefficient	*p*-value
**Anti-dsDNA IgG**	21	-0.02	0.920
**Anti-MDA IgM**	21	-0.40	0.070
**Anti-PC IgM**	21	-0.17	0.454
**BAFF**	20	0.03	0.885
**C3c**	21	-0.18	0.440
**C4**	21	-0.16	0.493
**CCL4**	14	-0.06	0.852
**CCL8**	14	-0.27	0.358
**CCL13**	14	0.39	0.169
**CCL17**	14	0.05	0.876
**CRP**	15	0.04	0.889
**CXCL10**	14	0.55	**0.041**
**CXCL13**	14	0.89	**<0.001**
**FasL**	14	0.29	0.311
**GDF15**	14	0.16	0.573
**IL-1R2**	14	-0.49	0.078
**IL-1RA**	14	0.07	0.817
**IL-6**	20	0.01	0.975
**MMP-2**	15	-0.56	**0.031**
**MMP-8**	14	-0.40	0.154
**TGF-ß**	20	0.21	0.369
**TNF-α**	20	0.38	0.101
**TNFR1**	14	-0.18	0.543

Spearman's rank correlation coefficients of correlations between levels of different serological markers and Z-scores of the cell cycle gene module. The total number of patients with available data is indicated. Statistically significant *p*-values are in bold. Only comparisons with sufficient numbers of observations (n≥10) are included.

anti-dsDNA: antibodies against double-stranded DNA; BAFF: B cell activating factor belonging to the tumour necrosis factor family; C3c: complement component 3c; C4: complement component 4; CCL4: C-C motif chemokine ligand 4; CCL8: C-C motif chemokine ligand 8; CCL13: C-C motif chemokine ligand 13; CCL17: C-C motif chemokine ligand 17; CNS: central nervous system; CRP: C-reactive protein; CXCL10: CXC motif chemokine ligand 10; CXCL13: CXC motif chemokine ligand 13; FasL: Fas ligand; GDF15: growth differentiation factor 15; Ig: immunoglobulin; IL-1R2: interleukin 1 receptor type 2; IL-1RA: interleukin 1 receptor type antagonist; IL-6: interleukin 6; MDA: malondialdehyde; MMP-2: matrix metalloproteinase 2; MMP-8: matrix metalloproteinase 8; PC: phosphorylcholine; TGF-β: transforming growth factor β; TNF-α: transforming growth factor α; TNFR1: tumour necrosis factor receptor 1.

Supplementary Table S7. Correlations between serological markers and z-scores of the "cytotoxic/NK cell" gene module in patients with active CNS lupus

Serological marker	n	Coefficient	*p*-value
**Anti-dsDNA IgG**	21	-0.34	0.136
**Anti-MDA IgM**	21	-0.12	0.590
**Anti-PC IgM**	21	-0.20	0.375
**BAFF**	20	0.20	0.402
**C3c**	21	0.26	0.263
**C4**	21	0.35	0.124
**CCL4**	14	-0.01	0.970
**CCL8**	14	0.17	0.563
**CCL13**	14	-0.04	0.887
**CCL17**	14	0.09	0.771
**CRP**	15	0.52	0.044
**CXCL10**	14	-0.30	0.303
**CXCL13**	14	-0.33	0.253
**FasL**	14	0.20	0.493
**GDF15**	14	0.03	0.911
**IL-1R2**	14	0.34	0.233
**IL-1RA**	14	-0.32	0.267
**IL-6**	20	0.29	0.222
**MMP-2**	15	0.52	0.044
**MMP-8**	14	-0.13	0.670
**TGF-ß**	20	-0.10	0.673
**TNF-α**	20	0.20	0.391
**TNFR1**	14	0.26	0.375

Spearman's rank correlation coefficients of correlations between levels of different serological markers and Z-scores of the cytotoxic/NK cell gene module. The total number of patients with available data is indicated. Statistically significant *p*-values are in bold. Only comparisons with sufficient numbers of observations (n≥10) are included.

anti-dsDNA: antibodies against double-stranded DNA; BAFF: B cell activating factor belonging to the tumour necrosis factor family; C3c: complement component 3c; C4: complement component 4; CCL4: C-C motif chemokine ligand 4; CCL8: C-C motif chemokine ligand 8; CCL13: C-C motif chemokine ligand 13; CCL17: C-C motif chemokine ligand 17; CNS: central nervous system; CRP: C-reactive protein; CXCL10: CXC motif chemokine ligand 10; CXCL13: CXC motif chemokine ligand 13; FasL: Fas ligand; GDF15: growth differentiation factor 15; Ig: immunoglobulin; IL-1R2: interleukin 1 receptor type 2; IL-1RA: interleukin 1 receptor type antagonist; IL-6: interleukin 6; MDA: malondialdehyde; MMP-2: matrix metalloproteinase 2; MMP-8: matrix metalloproteinase 8; NK: natural killer; PC: phosphorylcholine; TGF-β: transforming growth factor β; TNF-α: transforming growth factor α; TNFR1: tumour necrosis factor receptor 1.

Supplementary Table S8. Correlations between serological markers and z-scores of the "enriched for ubiquitination" gene module in patients with active CNS lupus

Serological marker	n	Coefficient	*p*-value
**Anti-dsDNA IgG**	21	-0.19	0.418
**Anti-MDA IgM**	21	0.08	0.724
**Anti-PC IgM**	21	0.02	0.920
**BAFF**	20	-0.07	0.777
**C3c**	21	0.42	0.055
**C4**	21	0.30	0.186
**CCL4**	14	0.40	0.161
**CCL8**	14	0.29	0.318
**CCL13**	14	0.34	0.233
**CCL17**	14	0.00	0.994
**CRP**	15	0.39	0.147
**CXCL10**	14	-0.30	0.296
**CXCL13**	14	-0.45	0.110
**FasL**	14	-0.33	0.253
**GDF15**	14	0.46	0.098
**IL-1R2**	14	0.56	**0.039**
**IL-1RA**	14	-0.13	0.648
**IL-6**	20	0.28	0.230
**MMP-2**	15	0.53	**0.041**
**MMP-8**	14	-0.02	0.946
**TGF-ß**	20	-0.09	0.719
**TNF-α**	20	0.00	0.995
**TNFR1**	14	0.15	0.615

Spearman's rank correlation coefficients of correlations between levels of different serological markers and Z-scores of the enriched for ubiquitination gene module. The total number of patients with available data is indicated. Statistically significant *p*-values are in bold. Only comparisons with sufficient numbers of observations (n≥10) are included.

anti-dsDNA: antibodies against double-stranded DNA; BAFF: B cell activating factor belonging to the tumour necrosis factor family; C3c: complement component 3c; C4: complement component 4; CCL4: C-C motif chemokine ligand 4; CCL8: C-C motif chemokine ligand 8; CCL13: C-C motif chemokine ligand 13; CCL17: C-C motif chemokine ligand 17; CNS: central nervous system; CRP: C-reactive protein; CXCL10: CXC motif chemokine ligand 10; CXCL13: CXC motif chemokine ligand 13; FasL: Fas ligand; GDF15: growth differentiation factor 15; Ig: immunoglobulin; IL-1R2: interleukin 1 receptor type 2; IL-1RA: interleukin 1 receptor type antagonist; IL-6: interleukin 6; MDA: malondialdehyde; MMP-2: matrix metalloproteinase 2; MMP-8: matrix metalloproteinase 8; PC: phosphorylcholine; TGF-β: transforming growth factor β; TNF-α: transforming growth factor α; TNFR1: tumour necrosis factor receptor 1.

Supplementary Table S9. Correlations between serological markers and z-scores of the "enriched in nuclear pore complex interacting proteins" gene module in patients with active CNS lupus

Serological marker	n	Coefficient	*p*-value
**Anti-dsDNA IgG**	21	0.10	0.653
**Anti-MDA IgM**	21	0.12	0.606
**Anti-PC IgM**	21	0.38	0.088
**BAFF**	20	-0.30	0.195
**C3c**	21	-0.38	0.089
**C4**	21	-0.25	0.281
**CCL4**	14	-0.15	0.620
**CCL8**	14	0.36	0.208
**CCL13**	14	-0.10	0.725
**CCL17**	14	0.04	0.899
**CRP**	15	0.02	0.950
**CXCL10**	14	0.30	0.296
**CXCL13**	14	-0.05	0.852
**FasL**	14	0.15	0.615
**GDF15**	14	-0.27	0.350
**IL-1R2**	14	-0.23	0.436
**IL-1RA**	14	0.36	0.208
**IL-6**	20	0.01	0.970
**MMP-2**	15	0.26	0.355
**MMP-8**	14	-0.06	0.829
**TGF-ß**	20	-0.45	**0.048**
**TNF-α**	20	-0.19	0.413
**TNFR1**	14	-0.06	0.840

Spearman's rank correlation coefficients of correlations between levels of different serological markers and Z-scores of the enriched in nuclear pore complex interacting proteins gene module. The total number of patients with available data is indicated. Statistically significant *p*-values are in bold. Only comparisons with sufficient numbers of observations (n≥10) are included.

anti-dsDNA: antibodies against double-stranded DNA; BAFF: B cell activating factor belonging to the tumour necrosis factor family; C3c: complement component 3c; C4: complement component 4; CCL4: C-C motif chemokine ligand 4; CCL8: C-C motif chemokine ligand 8; CCL13: C-C motif chemokine ligand 13; CCL17: C-C motif chemokine ligand 17; CNS: central nervous system; CRP: C-reactive protein; CXCL10: CXC motif chemokine ligand 10; CXCL13: CXC motif chemokine ligand 13; FasL: Fas ligand; GDF15: growth differentiation factor 15; Ig: immunoglobulin; IL-1R2: interleukin 1 receptor type 2; IL-1RA: interleukin 1 receptor type antagonist; IL-6: interleukin 6; MDA: malondialdehyde; MMP-2: matrix metalloproteinase 2; MMP-8: matrix metalloproteinase 8; PC: phosphorylcholine; TGF-β: transforming growth factor β; TNF-α: transforming growth factor α; TNFR1: tumour necrosis factor receptor 1.

Supplementary Table S10. Correlations between serological markers and z-scores of the "erythrocytes" gene module in patients with active CNS lupus

Serological marker	n	Coefficient	*p*-value
**Anti-dsDNA IgG**	21	-0.21	0.366
**Anti-MDA IgM**	21	0.10	0.666
**Anti-PC IgM**	21	0.29	0.195
**BAFF**	20	-0.05	0.850
**C3c**	21	-0.19	0.397
**C4**	21	-0.13	0.567
**CCL4**	14	0.08	0.794
**CCL8**	14	0.38	0.180
**CCL13**	14	-0.08	0.794
**CCL17**	14	-0.24	0.418
**CRP**	15	0.30	0.283
**CXCL10**	14	0.33	0.253
**CXCL13**	14	0.35	0.215
**FasL**	14	-0.03	0.923
**GDF15**	14	0.12	0.692
**IL-1R2**	14	-0.17	0.563
**IL-1RA**	14	-0.01	0.982
**IL-6**	20	0.27	0.248
**MMP-2**	15	0.39	0.152
**MMP-8**	14	-0.49	0.078
**TGF-ß**	20	-0.41	0.076
**TNF-α**	20	0.05	0.821
**TNFR1**	14	-0.16	0.594

Spearman's rank correlation coefficients of correlations between levels of different serological markers and Z-scores of the erythrocytes gene module. The total number of patients with available data is indicated. Statistically significant *p*-values are in bold. Only comparisons with sufficient numbers of observations (n≥10) are included.

anti-dsDNA: antibodies against double-stranded DNA; BAFF: B cell activating factor belonging to the tumour necrosis factor family; C3c: complement component 3c; C4: complement component 4; CCL4: C-C motif chemokine ligand 4; CCL8: C-C motif chemokine ligand 8; CCL13: C-C motif chemokine ligand 13; CCL17: C-C motif chemokine ligand 17; CNS: central nervous system; CRP: C-reactive protein; CXCL10: CXC motif chemokine ligand 10; CXCL13: CXC motif chemokine ligand 13; FasL: Fas ligand; GDF15: growth differentiation factor 15; Ig: immunoglobulin; IL-1R2: interleukin 1 receptor type 2; IL-1RA: interleukin 1 receptor type antagonist; IL-6: interleukin 6; MDA: malondialdehyde; MMP-2: matrix metalloproteinase 2; MMP-8: matrix metalloproteinase 8; PC: phosphorylcholine; TGF-β: transforming growth factor β; TNF-α: transforming growth factor α; TNFR1: tumour necrosis factor receptor 1.

Supplementary Table S11. Correlations between serological markers and z-scores of the "G protein coupled receptors cluster" gene module in patients with active CNS lupus

Serological marker	n	Coefficient	*p*-value
**Anti-dsDNA IgG**	21	-0.03	0.881
**Anti-MDA IgM**	21	0.13	0.567
**Anti-PC IgM**	21	0.12	0.602
**BAFF**	20	0.02	0.930
**C3c**	21	-0.16	0.496
**C4**	21	0.04	0.854
**CCL4**	14	-0.05	0.864
**CCL8**	14	-0.24	0.418
**CCL13**	14	-0.16	0.573
**CCL17**	14	0.38	0.180
**CRP**	15	-0.55	**0.032**
**CXCL10**	14	0.15	0.605
**CXCL13**	14	0.12	0.681
**FasL**	14	0.00	0.994
**GDF15**	14	-0.21	0.464
**IL-1R2**	14	-0.04	0.887
**IL-1RA**	14	-0.03	0.923
**IL-6**	20	-0.12	0.609
**MMP-2**	15	-0.04	0.899
**MMP-8**	14	0.46	0.098
**TGF-ß**	20	-0.21	0.376
**TNF-α**	20	-0.17	0.486
**TNFR1**	14	-0.03	0.923

Spearman's rank correlation coefficients of correlations between levels of different serological markers and Z-scores of the G protein coupled receptors cluster gene module. The total number of patients with available data is indicated. Statistically significant *p*-values are in bold. Only comparisons with sufficient numbers of observations (n≥10) are included.

anti-dsDNA: antibodies against double-stranded DNA; BAFF: B cell activating factor belonging to the tumour necrosis factor family; C3c: complement component 3c; C4: complement component 4; CCL4: C-C motif chemokine ligand 4; CCL8: C-C motif chemokine ligand 8; CCL13: C-C motif chemokine ligand 13; CCL17: C-C motif chemokine ligand 17; CNS: central nervous system; CRP: C-reactive protein; CXCL10: CXC motif chemokine ligand 10; CXCL13: CXC motif chemokine ligand 13; FasL: Fas ligand; GDF15: growth differentiation factor 15; Ig: immunoglobulin; IL-1R2: interleukin 1 receptor type 2; IL-1RA: interleukin 1 receptor type antagonist; IL-6: interleukin 6; MDA: malondialdehyde; MMP-2: matrix metalloproteinase 2; MMP-8: matrix metalloproteinase 8; PC: phosphorylcholine; TGF-β: transforming growth factor β; TNF-α: transforming growth factor α; TNFR1: tumour necrosis factor receptor 1.

Supplementary Table S12. Correlations between serological markers and z-scores of the "inflammation (II)" gene module in patients with active CNS lupus

Serological marker	n	Coefficient	*p*-value
**Anti-dsDNA IgG**	21	0.13	0.569
**Anti-MDA IgM**	21	0.18	0.434
**Anti-PC IgM**	21	0.28	0.225
**BAFF**	20	-0.06	0.811
**C3c**	21	-0.41	0.068
**C4**	21	-0.39	0.079
**CCL4**	14	-0.09	0.753
**CCL8**	14	0.13	0.648
**CCL13**	14	-0.09	0.771
**CCL17**	14	0.23	0.436
**CRP**	15	-0.18	0.516
**CXCL10**	14	0.36	0.203
**CXCL13**	14	0.13	0.670
**FasL**	14	-0.02	0.958
**GDF15**	14	-0.19	0.513
**IL-1R2**	14	-0.41	0.149
**IL-1RA**	14	0.34	0.233
**IL-6**	20	-0.07	0.767
**MMP-2**	15	-0.06	0.820
**MMP-8**	14	0.06	0.829
**TGF-ß**	20	-0.33	0.156
**TNF-α**	20	-0.07	0.782
**TNFR1**	14	-0.22	0.455

Spearman's rank correlation coefficients of correlations between levels of different serological markers and Z-scores of the inflammation (II) gene module. The total number of patients with available data is indicated. Statistically significant *p*-values are in bold. Only comparisons with sufficient numbers of observations (n≥10) are included.

anti-dsDNA: antibodies against double-stranded DNA; BAFF: B cell activating factor belonging to the tumour necrosis factor family; C3c: complement component 3c; C4: complement component 4; CCL4: C-C motif chemokine ligand 4; CCL8: C-C motif chemokine ligand 8; CCL13: C-C motif chemokine ligand 13; CCL17: C-C motif chemokine ligand 17; CNS: central nervous system; CRP: C-reactive protein; CXCL10: CXC motif chemokine ligand 10; CXCL13: CXC motif chemokine ligand 13; FasL: Fas ligand; GDF15: growth differentiation factor 15; Ig: immunoglobulin; IL-1R2: interleukin 1 receptor type 2; IL-1RA: interleukin 1 receptor type antagonist; IL-6: interleukin 6; MDA: malondialdehyde; MMP-2: matrix metalloproteinase 2; MMP-8: matrix metalloproteinase 8; PC: phosphorylcholine; TGF-β: transforming growth factor β; TNF-α: transforming growth factor α; TNFR1: tumour necrosis factor receptor 1.

Supplementary Table S13. Correlations between serological markers and z-scores of the "inflammation (IV)" gene module in patients with active CNS lupus

Serological marker	n	Coefficient	*p*-value
**Anti-dsDNA IgG**	21	-0.03	0.902
**Anti-MDA IgM**	21	0.15	0.529
**Anti-PC IgM**	21	-0.13	0.563
**BAFF**	20	0.15	0.535
**C3c**	21	0.52	**0.016**
**C4**	21	0.29	0.199
**CCL4**	14	0.72	**0.004**
**CCL8**	14	0.16	0.584
**CCL13**	14	0.19	0.523
**CCL17**	14	0.15	0.605
**CRP**	15	0.39	0.156
**CXCL10**	14	-0.06	0.829
**CXCL13**	14	-0.26	0.366
**FasL**	14	-0.83	**<0.001**
**GDF15**	14	0.74	**0.003**
**IL-1R2**	14	0.34	0.240
**IL-1RA**	14	-0.07	0.805
**IL-6**	20	0.40	0.082
**MMP-2**	15	0.42	0.114
**MMP-8**	14	0.21	0.464
**TGF-ß**	20	-0.38	0.099
**TNF-α**	20	0.06	0.801
**TNFR1**	14	0.25	0.392

Spearman's rank correlation coefficients of correlations between levels of different serological markers and Z-scores of the inflammation (IV) gene module. The total number of patients with available data is indicated. Statistically significant *p*-values are in bold. Only comparisons with sufficient numbers of observations (n≥10) are included.

anti-dsDNA: antibodies against double-stranded DNA; BAFF: B cell activating factor belonging to the tumour necrosis factor family; C3c: complement component 3c; C4: complement component 4; CCL4: C-C motif chemokine ligand 4; CCL8: C-C motif chemokine ligand 8; CCL13: C-C motif chemokine ligand 13; CCL17: C-C motif chemokine ligand 17; CNS: central nervous system; CRP: C-reactive protein; CXCL10: CXC motif chemokine ligand 10; CXCL13: CXC motif chemokine ligand 13; FasL: Fas ligand; GDF15: growth differentiation factor 15; Ig: immunoglobulin; IL-1R2: interleukin 1 receptor type 2; IL-1RA: interleukin 1 receptor type antagonist; IL-6: interleukin 6; MDA: malondialdehyde; MMP-2: matrix metalloproteinase 2; MMP-8: matrix metalloproteinase 8; PC: phosphorylcholine; TGF-β: transforming growth factor β; TNF-α: transforming growth factor α; TNFR1: tumour necrosis factor receptor 1.

Supplementary Table S14. Correlations between serological markers and z-scores of the "inflammation (VI)" gene module in patients with active CNS lupus

Serological marker	n	Coefficient	*p*-value
**Anti-dsDNA IgG**	21	0.25	0.267
**Anti-MDA IgM**	21	-0.47	0.032
**Anti-PC IgM**	21	-0.26	0.258
**BAFF**	20	0.27	0.246
**C3c**	21	-0.16	0.482
**C4**	21	-0.23	0.308
**CCL4**	14	-0.56	**0.038**
**CCL8**	14	-0.22	0.455
**CCL13**	14	-0.13	0.648
**CCL17**	14	-0.39	0.164
**CRP**	15	0.19	0.491
**CXCL10**	14	0.16	0.594
**CXCL13**	14	0.47	0.091
**FasL**	14	0.56	**0.037**
**GDF15**	14	-0.36	0.208
**IL-1R2**	14	-0.51	0.061
**IL-1RA**	14	0.05	0.852
**IL-6**	20	0.13	0.578
**MMP-2**	15	-0.06	0.840
**MMP-8**	14	-0.31	0.274
**TGF-ß**	20	0.07	0.772
**TNF-α**	20	0.38	0.098
**TNFR1**	14	-0.01	0.982

Spearman's rank correlation coefficients of correlations between levels of different serological markers and Z-scores of the inflammation (VI) gene module. The total number of patients with available data is indicated. Statistically significant *p*-values are in bold. Only comparisons with sufficient numbers of observations ($n \geq 10$) are included.

anti-dsDNA: antibodies against double-stranded DNA; BAFF: B cell activating factor belonging to the tumour necrosis factor family; C3c: complement component 3c; C4: complement component 4; CCL4: C-C motif chemokine ligand 4; CCL8: C-C motif chemokine ligand 8; CCL13: C-C motif chemokine ligand 13; CCL17: C-C motif chemokine ligand 17; CNS: central nervous system; CRP: C-reactive protein; CXCL10: CXC motif chemokine ligand 10; CXCL13: CXC motif chemokine ligand 13; FasL: Fas ligand; GDF15: growth differentiation factor 15; Ig: immunoglobulin; IL-1R2: interleukin 1 receptor type 2; IL-1RA: interleukin 1 receptor type antagonist; IL-6: interleukin 6; MDA: malondialdehyde; MMP-2: matrix metalloproteinase 2; MMP-8: matrix metalloproteinase 8; PC: phosphorylcholine; TGF-β: transforming growth factor β; TNF-α: transforming growth factor α; TNFR1: tumour necrosis factor receptor 1.

Supplementary Table S15. Correlations between serological markers and z-scores of the "inositol phosphate metabolism" gene module in patients with active CNS lupus

Serological marker	n	Coefficient	*p*-value
**Anti-dsDNA IgG**	21	-0.1	0.655
**Anti-MDA IgM**	21	0.18	0.434
**Anti-PC IgM**	21	0.09	0.699
**BAFF**	20	0.06	0.806
**C3c**	21	0.05	0.845
**C4**	21	0.03	0.902
**CCL4**	14	0.56	**0.038**
**CCL8**	14	0.53	**0.049**
**CCL13**	14	0.47	0.091
**CCL17**	14	0.23	0.436
**CRP**	15	0.19	0.508
**CXCL10**	14	0.42	0.140
**CXCL13**	14	-0.05	0.876
**FasL**	14	-0.38	0.185
**GDF15**	14	0.56	**0.037**
**IL-1R2**	14	0.02	0.935
**IL-1RA**	14	0.31	0.288
**IL-6**	20	0.34	0.148
**MMP-2**	15	0.60	**0.018**
**MMP-8**	14	-0.09	0.759
**TGF-ß**	20	-0.41	0.076
**TNF-α**	20	0.05	0.840
**TNFR1**	14	0.05	0.864

Spearman's rank correlation coefficients of correlations between levels of different serological markers and Z-scores of the inositol phosphate metabolism gene module. The total number of patients with available data is indicated. Statistically significant *p*-values are in bold. Only comparisons with sufficient numbers of observations (n≥10) are included.

anti-dsDNA: antibodies against double-stranded DNA; BAFF: B cell activating factor belonging to the tumour necrosis factor family; C3c: complement component 3c; C4: complement component 4; CCL4: C-C motif chemokine ligand 4; CCL8: C-C motif chemokine ligand 8; CCL13: C-C motif chemokine ligand 13; CCL17: C-C motif chemokine ligand 17; CNS: central nervous system; CRP: C-reactive protein; CXCL10: CXC motif chemokine ligand 10; CXCL13: CXC motif chemokine ligand 13; FasL: Fas ligand; GDF15: growth differentiation factor 15; Ig: immunoglobulin; IL-1R2: interleukin 1 receptor type 2; IL-1RA: interleukin 1 receptor type antagonist; IL-6: interleukin 6; MDA: malondialdehyde; MMP-2: matrix metalloproteinase 2; MMP-8: matrix metalloproteinase 8; PC: phosphorylcholine; TGF-β: transforming growth factor β; TNF-α: transforming growth factor α; TNFR1: tumour necrosis factor receptor 1.

Supplementary Table S16. Correlations between serological markers and z-scores of the "interferon" gene module in patients with active CNS lupus

Serological marker	n	Coefficient	*p*-value
**Anti-dsDNA IgG**	21	0.09	0.693
**Anti-MDA IgM**	21	0.13	0.563
**Anti-PC IgM**	21	0.06	0.780
**BAFF**	20	0.11	0.636
**C3c**	21	-0.11	0.646
**C4**	21	-0.38	0.090
**CCL4**	14	-0.02	0.946
**CCL8**	14	0.34	0.233
**CCL13**	14	0.26	0.366
**CCL17**	14	0.09	0.759
**CRP**	15	0.22	0.435
**CXCL10**	14	0.68	**0.008**
**CXCL13**	14	0.41	0.149
**FasL**	14	0.24	0.418
**GDF15**	14	0.12	0.692
**IL-1R2**	14	-0.55	**0.043**
**IL-1RA**	14	0.67	**0.009**
**IL-6**	20	0.27	0.246
**MMP-2**	15	-0.11	0.694
**MMP-8**	14	-0.59	**0.026**
**TGF-ß**	20	-0.08	0.743
**TNF-α**	20	0.30	0.195
**TNFR1**	14	-0.29	0.311

Spearman's rank correlation coefficients of correlations between levels of different serological markers and Z-scores of the interferon gene module. The total number of patients with available data is indicated. Statistically significant *p*-values are in bold. Only comparisons with sufficient numbers of observations (n≥10) are included.

anti-dsDNA: antibodies against double-stranded DNA; BAFF: B cell activating factor belonging to the tumour necrosis factor family; C3c: complement component 3c; C4: complement component 4; CCL4: C-C motif chemokine ligand 4; CCL8: C-C motif chemokine ligand 8; CCL13: C-C motif chemokine ligand 13; CCL17: C-C motif chemokine ligand 17; CNS: central nervous system; CRP: C-reactive protein; CXCL10: CXC motif chemokine ligand 10; CXCL13: CXC motif chemokine ligand 13; FasL: Fas ligand; GDF15: growth differentiation factor 15; Ig: immunoglobulin; IL-1R2: interleukin 1 receptor type 2; IL-1RA: interleukin 1 receptor type antagonist; IL-6: interleukin 6; MDA: malondialdehyde; MMP-2: matrix metalloproteinase 2; MMP-8: matrix metalloproteinase 8; PC: phosphorylcholine; TGF-β: transforming growth factor β; TNF-α: transforming growth factor α; TNFR1: tumour necrosis factor receptor 1.

Supplementary Table S17. Correlations between serological markers and z-scores of the "intracellular transport" gene module in patients with active CNS lupus

Serological marker	n	Coefficient	*p*-value
**Anti-dsDNA IgG**	21	-0.23	0.313
**Anti-MDA IgM**	21	-0.04	0.871
**Anti-PC IgM**	21	-0.07	0.771
**BAFF**	20	-0.13	0.578
**C3c**	21	0.17	0.471
**C4**	21	0.28	0.214
**CCL4**	14	0.2	0.488
**CCL8**	14	0.45	0.110
**CCL13**	14	0.28	0.326
**CCL17**	14	-0.08	0.794
**CRP**	15	0.05	0.869
**CXCL10**	14	-0.20	0.493
**CXCL13**	14	-0.30	0.296
**FasL**	14	-0.02	0.946
**GDF15**	14	0.28	0.326
**IL-1R2**	14	0.41	0.149
**IL-1RA**	14	-0.14	0.626
**IL-6**	20	0.25	0.286
**MMP-2**	15	0.42	0.118
**MMP-8**	14	-0.19	0.523
**TGF-ß**	20	-0.02	0.920
**TNF-α**	20	-0.02	0.945
**TNFR1**	14	0.20	0.483

Spearman's rank correlation coefficients of correlations between levels of different serological markers and Z-scores of the intracellular transport gene module. The total number of patients with available data is indicated. Statistically significant *p*-values are in bold. Only comparisons with sufficient numbers of observations (n≥10) are included.

anti-dsDNA: antibodies against double-stranded DNA; BAFF: B cell activating factor belonging to the tumour necrosis factor family; C3c: complement component 3c; C4: complement component 4; CCL4: C-C motif chemokine ligand 4; CCL8: C-C motif chemokine ligand 8; CCL13: C-C motif chemokine ligand 13; CCL17: C-C motif chemokine ligand 17; CNS: central nervous system; CRP: C-reactive protein; CXCL10: CXC motif chemokine ligand 10; CXCL13: CXC motif chemokine ligand 13; FasL: Fas ligand; GDF15: growth differentiation factor 15; Ig: immunoglobulin; IL-1R2: interleukin 1 receptor type 2; IL-1RA: interleukin 1 receptor type antagonist; IL-6: interleukin 6; MDA: malondialdehyde; MMP-2: matrix metalloproteinase 2; MMP-8: matrix metalloproteinase 8; PC: phosphorylcholine; TGF-β: transforming growth factor β; TNF-α: transforming growth factor α; TNFR1: tumour necrosis factor receptor 1.

Supplementary Table S18. Correlations between serological markers and z-scores of the "mitochondrial cluster" gene module in patients with active CNS lupus

Serological marker	n	Coefficient	*p*-value
**Anti-dsDNA IgG**	21	-0.27	0.244
**Anti-MDA IgM**	21	-0.04	0.862
**Anti-PC IgM**	21	0.09	0.695
**BAFF**	20	-0.37	0.107
**C3c**	21	0.27	0.229
**C4**	21	0.46	**0.037**
**CCL4**	14	0.27	0.353
**CCL8**	14	0.31	0.274
**CCL13**	14	0.18	0.533
**CCL17**	14	-0.16	0.594
**CRP**	15	0.00	0.990
**CXCL10**	14	-0.43	0.122
**CXCL13**	14	-0.49	0.072
**FasL**	14	-0.16	0.573
**GDF15**	14	0.33	0.253
**IL-1R2**	14	0.65	**0.011**
**IL-1RA**	14	-0.24	0.401
**IL-6**	20	0.09	0.691
**MMP-2**	15	0.45	0.089
**MMP-8**	14	-0.06	0.840
**TGF-ß**	20	0.07	0.758
**TNF-α**	20	-0.29	0.212
**TNFR1**	14	0.31	0.288

Spearman's rank correlation coefficients of correlations between levels of different serological markers and Z-scores of the mitochondrial cluster gene module. The total number of patients with available data is indicated. Statistically significant *p*-values are in bold. Only comparisons with sufficient numbers of observations (n≥10) are included.

anti-dsDNA: antibodies against double-stranded DNA; BAFF: B cell activating factor belonging to the tumour necrosis factor family; C3c: complement component 3c; C4: complement component 4; CCL4: C-C motif chemokine ligand 4; CCL8: C-C motif chemokine ligand 8; CCL13: C-C motif chemokine ligand 13; CCL17: C-C motif chemokine ligand 17; CNS: central nervous system; CRP: C-reactive protein; CXCL10: CXC motif chemokine ligand 10; CXCL13: CXC motif chemokine ligand 13; FasL: Fas ligand; GDF15: growth differentiation factor 15; Ig: immunoglobulin; IL-1R2: interleukin 1 receptor type 2; IL-1RA: interleukin 1 receptor type antagonist; IL-6: interleukin 6; MDA: malondialdehyde; MMP-2: matrix metalloproteinase 2; MMP-8: matrix metalloproteinase 8; PC: phosphorylcholine; TGF-β: transforming growth factor β; TNF-α: transforming growth factor α; TNFR1: tumour necrosis factor receptor 1.

Supplementary Table S19. Correlations between serological markers and z-scores of the "myeloid cell enriched receptors and transporters" gene module in patients with active CNS lupus

Serological marker	n	Coefficient	*p*-value
**Anti-dsDNA IgG**	21	0.02	0.936
**Anti-MDA IgM**	21	-0.37	0.095
**Anti-PC IgM**	21	-0.24	0.286
**BAFF**	20	-0.22	0.352
**C3c**	21	-0.12	0.618
**C4**	21	-0.03	0.884
**CCL4**	14	0.43	0.126
**CCL8**	14	0.05	0.876
**CCL13**	14	0.58	**0.030**
**CCL17**	14	-0.18	0.543
**CRP**	15	-0.12	0.657
**CXCL10**	14	0.09	0.748
**CXCL13**	14	0.40	0.159
**FasL**	14	-0.38	0.175
**GDF15**	14	0.60	**0.025**
**IL-1R2**	14	0.24	0.401
**IL-1RA**	14	-0.27	0.342
**IL-6**	20	0.28	0.230
**MMP-2**	15	0.35	0.201
**MMP-8**	14	-0.01	0.982
**TGF-ß**	20	-0.17	0.478
**TNF-α**	20	0.12	0.622
**TNFR1**	14	0.31	0.274

Spearman's rank correlation coefficients of correlations between levels of different serological markers and Z-scores of the myeloid cell enriched receptors and transporters gene module. The total number of patients with available data is indicated. Statistically significant *p*-values are in bold. Only comparisons with sufficient numbers of observations (n≥10) are included.

anti-dsDNA: antibodies against double-stranded DNA; BAFF: B cell activating factor belonging to the tumour necrosis factor family; C3c: complement component 3c; C4: complement component 4; CCL4: C-C motif chemokine ligand 4; CCL8: C-C motif chemokine ligand 8; CCL13: C-C motif chemokine ligand 13; CCL17: C-C motif chemokine ligand 17; CNS: central nervous system; CRP: C-reactive protein; CXCL10: CXC motif chemokine ligand 10; CXCL13: CXC motif chemokine ligand 13; FasL: Fas ligand; GDF15: growth differentiation factor 15; Ig: immunoglobulin; IL-1R2: interleukin 1 receptor type 2; IL-1RA: interleukin 1 receptor type antagonist; IL-6: interleukin 6; MDA: malondialdehyde; MMP-2: matrix metalloproteinase 2; MMP-8: matrix metalloproteinase 8; PC: phosphorylcholine; TGF-β: transforming growth factor β; TNF-α: transforming growth factor α; TNFR1: tumour necrosis factor receptor 1.

Supplementary Table S20. Correlations between serological markers and z-scores of the "Myeloid, dendritic cell activation via NFkB (I)" gene module in patients with active CNS lupus

Serological marker	n	Coefficient	*p*-value
**Anti-dsDNA IgG**	21	0.08	0.743
**Anti-MDA IgM**	21	0.03	0.884
**Anti-PC IgM**	21	0.42	0.058
**BAFF**	20	-0.26	0.268
**C3c**	21	-0.29	0.203
**C4**	21	-0.05	0.836
**CCL4**	14	-0.30	0.295
**CCL8**	14	0.44	0.114
**CCL13**	14	-0.05	0.852
**CCL17**	14	-0.29	0.311
**CRP**	15	0.03	0.909
**CXCL10**	14	-0.09	0.748
**CXCL13**	14	-0.38	0.185
**FasL**	14	0.32	0.267
**GDF15**	14	-0.39	0.169
**IL-1R2**	14	0.04	0.887
**IL-1RA**	14	0.22	0.446
**IL-6**	20	0.09	0.705
**MMP-2**	15	0.50	0.056
**MMP-8**	14	-0.17	0.563
**TGF-ß**	20	-0.09	0.705
**TNF-α**	20	-0.27	0.257
**TNFR1**	14	-0.06	0.829

Spearman's rank correlation coefficients of correlations between levels of different serological markers and Z-scores of the myeloid, dendritic cell activation via NFkB (I) gene module. The total number of patients with available data is indicated. Statistically significant *p*-values are in bold. Only comparisons with sufficient numbers of observations (n≥10) are included.

anti-dsDNA: antibodies against double-stranded DNA; BAFF: B cell activating factor belonging to the tumour necrosis factor family; C3c: complement component 3c; C4: complement component 4; CCL4: C-C motif chemokine ligand 4; CCL8: C-C motif chemokine ligand 8; CCL13: C-C motif chemokine ligand 13; CCL17: C-C motif chemokine ligand 17; CNS: central nervous system; CRP: C-reactive protein; CXCL10: CXC motif chemokine ligand 10; CXCL13: CXC motif chemokine ligand 13; FasL: Fas ligand; GDF15: growth differentiation factor 15; Ig: immunoglobulin; IL-1R2: interleukin 1 receptor type 2; IL-1RA: interleukin 1 receptor type antagonist; IL-6: interleukin 6; MDA: malondialdehyde; MMP-2: matrix metalloproteinase 2; MMP-8: matrix metalloproteinase 8; NFkB: Nuclear Factor Kappa B; PC: phosphorylcholine; TGF-β: transforming growth factor β; TNF-α: transforming growth factor α; TNFR1: tumour necrosis factor receptor 1.

Supplementary Table S21. Correlations between serological markers and z-scores of the "plasma cells, immunoglobulins" gene module in patients with active CNS lupus

Serological marker	n	Coefficient	*p*-value
**Anti-dsDNA IgG**	21	0.14	0.534
**Anti-MDA IgM**	21	-0.17	0.454
**Anti-PC IgM**	21	0.26	0.246
**BAFF**	20	-0.39	0.088
**C3c**	21	-0.14	0.548
**C4**	21	0.10	0.662
**CCL4**	14	-0.06	0.840
**CCL8**	14	-0.41	0.149
**CCL13**	14	-0.11	0.703
**CCL17**	14	0.00	0.994
**CRP**	15	-0.66	**0.007**
**CXCL10**	14	-0.25	0.392
**CXCL13**	14	0.13	0.659
**FasL**	14	-0.37	0.191
**GDF15**	14	-0.10	0.725
**IL-1R2**	14	-0.06	0.829
**IL-1RA**	14	-0.31	0.274
**IL-6**	20	-0.14	0.565
**MMP-2**	15	-0.37	0.173
**MMP-8**	14	0.49	0.075
**TGF-ß**	20	0.18	0.450
**TNF-α**	20	-0.27	0.243
**TNFR1**	14	-0.22	0.455

Spearman's rank correlation coefficients of correlations between levels of different serological markers and Z-scores of the plasma cells, immunoglobulins gene module. The total number of patients with available data is indicated. Statistically significant *p*-values are in bold. Only comparisons with sufficient numbers of observations (n≥10) are included.

anti-dsDNA: antibodies against double-stranded DNA; BAFF: B cell activating factor belonging to the tumour necrosis factor family; C3c: complement component 3c; C4: complement component 4; CCL4: C-C motif chemokine ligand 4; CCL8: C-C motif chemokine ligand 8; CCL13: C-C motif chemokine ligand 13; CCL17: C-C motif chemokine ligand 17; CNS: central nervous system; CRP: C-reactive protein; CXCL10: CXC motif chemokine ligand 10; CXCL13: CXC motif chemokine ligand 13; FasL: Fas ligand; GDF15: growth differentiation factor 15; Ig: immunoglobulin; IL-1R2: interleukin 1 receptor type 2; IL-1RA: interleukin 1 receptor type antagonist; IL-6: interleukin 6; MDA: malondialdehyde; MMP-2: matrix metalloproteinase 2; MMP-8: matrix metalloproteinase 8; PC: phosphorylcholine; TGF-β: transforming growth factor β; TNF-α: transforming growth factor α; TNFR1: tumour necrosis factor receptor 1.

Supplementary Table S22. Correlations between serological markers and z-scores of the "platelets" gene module in patients with active CNS lupus

Serological marker	n	Coefficient	*p*-value
**Anti-dsDNA IgG**	21	-0.06	0.793
**Anti-MDA IgM**	21	-0.09	0.683
**Anti-PC IgM**	21	-0.25	0.284
**BAFF**	20	0.51	0.023
**C3c**	21	0.47	**0.031**
**C4**	21	0.17	0.454
**CCL4**	14	-0.18	0.537
**CCL8**	14	-0.26	0.375
**CCL13**	14	0.08	0.782
**CCL17**	14	0.13	0.648
**CRP**	15	0.27	0.328
**CXCL10**	14	-0.05	0.876
**CXCL13**	14	0.39	0.164
**FasL**	14	0.31	0.288
**GDF15**	14	0.09	0.759
**IL-1R2**	14	-0.17	0.553
**IL-1RA**	14	-0.15	0.615
**IL-6**	20	0.48	**0.034**
**MMP-2**	15	-0.12	0.666
**MMP-8**	14	-0.33	0.253
**TGF-ß**	20	0.30	0.195
**TNF-α**	20	0.60	**0.005**
**TNFR1**	14	-0.10	0.737

Spearman's rank correlation coefficients of correlations between levels of different serological markers and Z-scores of the platelets gene module. The total number of patients with available data is indicated. Statistically significant *p*-values are in bold. Only comparisons with sufficient numbers of observations (n≥10) are included.

anti-dsDNA: antibodies against double-stranded DNA; BAFF: B cell activating factor belonging to the tumour necrosis factor family; C3c: complement component 3c; C4: complement component 4; CCL4: C-C motif chemokine ligand 4; CCL8: C-C motif chemokine ligand 8; CCL13: C-C motif chemokine ligand 13; CCL17: C-C motif chemokine ligand 17; CNS: central nervous system; CRP: C-reactive protein; CXCL10: CXC motif chemokine ligand 10; CXCL13: CXC motif chemokine ligand 13; FasL: Fas ligand; GDF15: growth differentiation factor 15; Ig: immunoglobulin; IL-1R2: interleukin 1 receptor type 2; IL-1RA: interleukin 1 receptor type antagonist; IL-6: interleukin 6; MDA: malondialdehyde; MMP-2: matrix metalloproteinase 2; MMP-8: matrix metalloproteinase 8; PC: phosphorylcholine; TGF-β: transforming growth factor β; TNF-α: transforming growth factor α; TNFR1: tumour necrosis factor receptor 1.

Supplementary Table S23. Correlations between serological markers and z-scores of the "regulation of transcription, transcription factors" gene module in patients with active CNS lupus

Serological marker	n	Coefficient	*p*-value
**Anti-dsDNA IgG**	21	-0.17	0.475
**Anti-MDA IgM**	21	0.35	0.115
**Anti-PC IgM**	21	0.59	0.005
**BAFF**	20	-0.39	0.086
**C3c**	21	-0.28	0.225
**C4**	21	-0.10	0.662
**CCL4**	14	0.31	0.273
**CCL8**	14	0.60	**0.025**
**CCL13**	14	0.15	0.615
**CCL17**	14	0.22	0.455
**CRP**	15	0.13	0.639
**CXCL10**	14	0.22	0.455
**CXCL13**	14	-0.45	0.110
**FasL**	14	-0.20	0.503
**GDF15**	14	0.09	0.771
**IL-1R2**	14	0.24	0.409
**IL-1RA**	14	0.31	0.288
**IL-6**	20	-0.04	0.880
**MMP-2**	15	0.54	**0.038**
**MMP-8**	14	0.08	0.782
**TGF-ß**	20	-0.54	**0.014**
**TNF-α**	20	-0.43	0.059
**TNFR1**	14	0.04	0.887

Spearman's rank correlation coefficients of correlations between levels of different serological markers and Z-scores of the regulation of transcription, transcription factors gene module. The total number of patients with available data is indicated. Statistically significant *p*-values are in bold. Only comparisons with sufficient numbers of observations (n≥10) are included.

anti-dsDNA: antibodies against double-stranded DNA; BAFF: B cell activating factor belonging to the tumour necrosis factor family; C3c: complement component 3c; C4: complement component 4; CCL4: C-C motif chemokine ligand 4; CCL8: C-C motif chemokine ligand 8; CCL13: C-C motif chemokine ligand 13; CCL17: C-C motif chemokine ligand 17; CNS: central nervous system; CRP: C-reactive protein; CXCL10: CXC motif chemokine ligand 10; CXCL13: CXC motif chemokine ligand 13; FasL: Fas ligand; GDF15: growth differentiation factor 15; Ig: immunoglobulin; IL-1R2: interleukin 1 receptor type 2; IL-1RA: interleukin 1 receptor type antagonist; IL-6: interleukin 6; MDA: malondialdehyde; MMP-2: matrix metalloproteinase 2; MMP-8: matrix metalloproteinase 8; PC: phosphorylcholine; TGF-β: transforming growth factor β; TNF-α: transforming growth factor α; TNFR1: tumour necrosis factor receptor 1.

Supplementary Table S24. Correlations between serological markers and z-scores of the "resting dendritic cell surface signature" gene module in patients with active CNS lupus

Serological marker	n	Coefficient	*p*-value
**Anti-dsDNA IgG**	21	-0.16	0.489
**Anti-MDA IgM**	21	-0.13	0.575
**Anti-PC IgM**	21	0.05	0.832
**BAFF**	20	-0.17	0.486
**C3c**	21	0.06	0.788
**C4**	21	0.30	0.192
**CCL4**	14	-0.21	0.464
**CCL8**	14	0.20	0.503
**CCL13**	14	-0.01	0.970
**CCL17**	14	-0.53	0.054
**CRP**	15	0.14	0.630
**CXCL10**	14	-0.45	0.102
**CXCL13**	14	-0.32	0.267
**FasL**	14	0.28	0.334
**GDF15**	14	-0.14	0.637
**IL-1R2**	14	0.32	0.260
**IL-1RA**	14	-0.22	0.446
**IL-6**	20	0.23	0.319
**MMP-2**	15	0.61	**0.016**
**MMP-8**	14	-0.24	0.409
**TGF-ß**	20	0.18	0.443
**TNF-α**	20	-0.10	0.686
**TNFR1**	14	0.11	0.703

Spearman's rank correlation coefficients of correlations between levels of different serological markers and Z-scores of the resting dendritic cell surface signature gene module. The total number of patients with available data is indicated. Statistically significant *p*-values are in bold. Only comparisons with sufficient numbers of observations (n≥10) are included.

anti-dsDNA: antibodies against double-stranded DNA; BAFF: B cell activating factor belonging to the tumour necrosis factor family; C3c: complement component 3c; C4: complement component 4; CCL4: C-C motif chemokine ligand 4; CCL8: C-C motif chemokine ligand 8; CCL13: C-C motif chemokine ligand 13; CCL17: C-C motif chemokine ligand 17; CNS: central nervous system; CRP: C-reactive protein; CXCL10: CXC motif chemokine ligand 10; CXCL13: CXC motif chemokine ligand 13; FasL: Fas ligand; GDF15: growth differentiation factor 15; Ig: immunoglobulin; IL-1R2: interleukin 1 receptor type 2; IL-1RA: interleukin 1 receptor type antagonist; IL-6: interleukin 6; MDA: malondialdehyde; MMP-2: matrix metalloproteinase 2; MMP-8: matrix metalloproteinase 8; PC: phosphorylcholine; TGF-β: transforming growth factor β; TNF-α: transforming growth factor α; TNFR1: tumour necrosis factor receptor 1.

Supplementary Table S25. Correlations between serological markers and z-scores of the "small GTPase mediated signal transduction" gene module in patients with active CNS lupus

Serological marker	n	Coefficient	*p*-value
**Anti-dsDNA IgG**	21	0.29	0.204
**Anti-MDA IgM**	21	-0.12	0.594
**Anti-PC IgM**	21	0.17	0.464
**BAFF**	20	0.05	0.826
**C3c**	21	-0.31	0.165
**C4**	21	-0.31	0.179
**CCL4**	14	-0.51	0.063
**CCL8**	14	-0.06	0.840
**CCL13**	14	-0.31	0.288
**CCL17**	14	-0.16	0.573
**CRP**	15	0.00	0.990
**CXCL10**	14	0.17	0.553
**CXCL13**	14	0.13	0.659
**FasL**	14	0.41	0.144
**GDF15**	14	-0.56	**0.037**
**IL-1R2**	14	-0.48	0.081
**IL-1RA**	14	0.25	0.383
**IL-6**	20	-0.09	0.719
**MMP-2**	15	-0.09	0.752
**MMP-8**	14	-0.06	0.840
**TGF-ß**	20	-0.12	0.600
**TNF-α**	20	-0.02	0.950
**TNFR1**	14	-0.16	0.573

Spearman's rank correlation coefficients of correlations between levels of different serological markers and Z-scores of the small GTPase mediated signal transduction gene module. The total number of patients with available data is indicated. Statistically significant *p*-values are in bold. Only comparisons with sufficient numbers of observations (n≥10) are included.

anti-dsDNA: antibodies against double-stranded DNA; BAFF: B cell activating factor belonging to the tumour necrosis factor family; C3c: complement component 3c; C4: complement component 4; CCL4: C-C motif chemokine ligand 4; CCL8: C-C motif chemokine ligand 8; CCL13: C-C motif chemokine ligand 13; CCL17: C-C motif chemokine ligand 17; CNS: central nervous system; CRP: C-reactive protein; CXCL10: CXC motif chemokine ligand 10; CXCL13: CXC motif chemokine ligand 13; FasL: Fas ligand; GDF15: growth differentiation factor 15; Ig: immunoglobulin; IL-1R2: interleukin 1 receptor type 2; IL-1RA: interleukin 1 receptor type antagonist; IL-6: interleukin 6; MDA: malondialdehyde; MMP-2: matrix metalloproteinase 2; MMP-8: matrix metalloproteinase 8; PC: phosphorylcholine; TGF-β: transforming growth factor β; TNF-α: transforming growth factor α; TNFR1: tumour necrosis factor receptor 1.

Supplementary Table S26. Correlations between serological markers and z-scores of the "spliceosome" gene module in patients with active CNS lupus

Serological marker	n	Coefficient	*p*-value
**Anti-dsDNA IgG**	21	-0.19	0.418
**Anti-MDA IgM**	21	0.23	0.325
**Anti-PC IgM**	21	0.24	0.294
**BAFF**	20	-0.35	0.126
**C3c**	21	0.43	0.050
**C4**	21	0.48	**0.029**
**CCL4**	14	0.30	0.291
**CCL8**	14	0.19	0.523
**CCL13**	14	-0.06	0.829
**CCL17**	14	-0.06	0.840
**CRP**	15	-0.22	0.435
**CXCL10**	14	-0.38	0.175
**CXCL13**	14	-0.64	**0.015**
**FasL**	14	-0.58	**0.029**
**GDF15**	14	0.32	0.260
**IL-1R2**	14	0.44	0.118
**IL-1RA**	14	-0.02	0.958
**IL-6**	20	0.04	0.875
**MMP-2**	15	0.43	0.108
**MMP-8**	14	0.25	0.383
**TGF-ß**	20	-0.11	0.645
**TNF-α**	20	-0.46	**0.041**
**TNFR1**	14	-0.06	0.840

Spearman's rank correlation coefficients of correlations between levels of different serological markers and Z-scores of the spliceosome gene module. The total number of patients with available data is indicated. Statistically significant *p*-values are in bold. Only comparisons with sufficient numbers of observations ($n \geq 10$) are included.

anti-dsDNA: antibodies against double-stranded DNA; BAFF: B cell activating factor belonging to the tumour necrosis factor family; C3c: complement component 3c; C4: complement component 4; CCL4: C-C motif chemokine ligand 4; CCL8: C-C motif chemokine ligand 8; CCL13: C-C motif chemokine ligand 13; CCL17: C-C motif chemokine ligand 17; CNS: central nervous system; CRP: C-reactive protein; CXCL10: CXC motif chemokine ligand 10; CXCL13: CXC motif chemokine ligand 13; FasL: Fas ligand; GDF15: growth differentiation factor 15; Ig: immunoglobulin; IL-1R2: interleukin 1 receptor type 2; IL-1RA: interleukin 1 receptor type antagonist; IL-6: interleukin 6; MDA: malondialdehyde; MMP-2: matrix metalloproteinase 2; MMP-8: matrix metalloproteinase 8; PC: phosphorylcholine; TGF-β: transforming growth factor β; TNF-α: transforming growth factor α; TNFR1: tumour necrosis factor receptor 1.

Supplementary Table S27. Correlations between serological markers and z-scores of the "T cell" gene module in patients with active CNS lupus

Serological marker	n	Coefficient	*p*-value
**Anti-dsDNA IgG**	21	-0.03	0.911
**Anti-MDA IgM**	21	0.10	0.658
**Anti-PC IgM**	21	0.46	0.037
**BAFF**	20	-0.47	0.038
**C3c**	21	-0.06	0.784
**C4**	21	0.18	0.447
**CCL4**	14	0.04	0.893
**CCL8**	14	0.49	0.072
**CCL13**	14	0.00	0.994
**CCL17**	14	-0.19	0.513
**CRP**	15	-0.21	0.459
**CXCL10**	14	-0.32	0.260
**CXCL13**	14	-0.73	0.003
**FasL**	14	-0.10	0.725
**GDF15**	14	-0.09	0.759
**IL-1R2**	14	0.48	0.085
**IL-1RA**	14	0.02	0.958
**IL-6**	20	-0.06	0.811
**MMP-2**	15	0.59	**0.022**
**MMP-8**	14	0.14	0.637
**TGF-ß**	20	-0.09	0.710
**TNF-α**	20	-0.48	**0.032**
**TNFR1**	14	0.13	0.659

Spearman's rank correlation coefficients of correlations between levels of different serological markers and Z-scores of the T cell gene module. The total number of patients with available data is indicated. Statistically significant *p*-values are in bold. Only comparisons with sufficient numbers of observations (n≥10) are included.

anti-dsDNA: antibodies against double-stranded DNA; BAFF: B cell activating factor belonging to the tumour necrosis factor family; C3c: complement component 3c; C4: complement component 4; CCL4: C-C motif chemokine ligand 4; CCL8: C-C motif chemokine ligand 8; CCL13: C-C motif chemokine ligand 13; CCL17: C-C motif chemokine ligand 17; CNS: central nervous system; CRP: C-reactive protein; CXCL10: CXC motif chemokine ligand 10; CXCL13: CXC motif chemokine ligand 13; FasL: Fas ligand; GDF15: growth differentiation factor 15; Ig: immunoglobulin; IL-1R2: interleukin 1 receptor type 2; IL-1RA: interleukin 1 receptor type antagonist; IL-6: interleukin 6; MDA: malondialdehyde; MMP-2: matrix metalloproteinase 2; MMP-8: matrix metalloproteinase 8; PC: phosphorylcholine; TGF-β: transforming growth factor β; TNF-α: transforming growth factor α; TNFR1: tumour necrosis factor receptor 1.

Supplementary Table S28. Z-scores of gene modules in patients with active CNS lupus and anti-chromatin positivity versus negative patients

Gene module	Anti-chromatin (+) n=12	Anti-chromatin (-) n=8	*p*-value
**B cell**	-0.85 (-1.81–-0.41)	-0.65 (-1.20–-0.02)	0.537
**Cell cycle**	0.37 (0.14–1.04)	0.30 (0.13–0.69)	0.939
**Cytotoxic/NK cell**	-0.53 (-1.25–-0.05)	-0.40 (-1.00–-0.08)	0.877
**Enriched for ubiquitination**	-0.04 (-0.23–0.28)	0.13 (-0.19–0.53)	0.487
**Enriched in nuclear pore complex interacting proteins**	-0.01 (-0.27–0.31)	-0.03 (-0.14–0.29)	0.817
**Erythrocytes**	-0.52 (-0.61–0.32)	-0.15 (-0.32–-0.01)	0.877
**G protein coupled receptors cluster**	-0.04 (-0.50–0.49)	0.41 (0.22–0.63)	0.355
**Inflammation II**	0.28 (0.00–0.66)	0.66 (0.39–0.78)	0.396
**Inflammation IV**	0.25 (0.01–0.44)	0.66 (0.27–0.97)	0.217
**Inflammation VI**	-0.01 (-0.22–0.17)	-0.22 (-0.50–-0.01)	0.143
**Inositol phosphate metabolism**	0.24 (-0.06–0.40)	0.42 (0.38–0.49)	0.105
**Interferon**	0.35 (0.02–1.12)	0.78 (0.45–0.92)	0.280
**Intracellular transport**	-0.13 (-0.44–0.25)	0.03 (-0.27–0.34)	0.589
**Mitochondrial cluster**	-0.42 (-0.86–0.10)	-0.22 (-0.88–0.11)	0.758
**Myeloid cell enriched receptors and transporters**	0.43 (0.19–0.70)	0.11 (-0.47–0.74)	0.589
**Myeloid, dendritic cell activation via NFkB (I)**	-0.31 (-0.78–0.06)	-0.64 (-0.97–-0.13)	0.643
**Plasma cells, immunoglobulins**	0.35 (-0.30–0.60)	-0.27 (-0.37–0.09)	0.316
**Platelets**	-0.16 (-0.64–0.24)	-0.15 (-0.59–-0.01)	1.000
**Regulation of transcription, transcription factors**	-0.07 (-0.47–0.32)	0.04 (-0.19–0.58)	0.355
**Resting dendritic cell surface signature**	-0.28 (-0.60–-0.12)	-0.38 (-0.89–-0.20)	0.440
**Small GTPase mediated signal transduction**	0.01 (-0.66–0.36)	-0.04 (-1.00–0.21)	0.355
**Spliceosome**	-0.29 (-0.71–0.42)	0.04 (-0.49–0.60)	0.247
**T cell**	-0.24 (-1.05–-0.09)	-0.23 (-1.19–0.15)	0.817

Data are presented as median (interquartile range). The total number of patients with available data is indicated. All *p*-values are derived from non-parametrical Mann-Whitney *U* tests. Statistically significant *p* values are in bold.

CNS: central nervous system; NFkB: Nuclear Factor Kappa B; NK: natural killer.

Supplementary Table S29. Z-scores of gene modules in patients with active CNS lupus and anti-dsDNA positivity versus negative patients

Gene module	Anti-dsDNA (+) n=4	Anti-dsDNA (-) n=17	*p*-value
**B cell**	-2.22 (-2.52–-1.82)	-0.56 (-1.12–-0.02)	**0.016**
**Cell cycle**	0.73 (0.35–1.04)	0.31 (0.15–0.84)	0.591
**Cytotoxic/NK cell**	-1.37 (-1.52–-1.15)	-0.25 (-0.63–0.22)	**0.020**
**Enriched for ubiquitination**	-0.31 (-0.58–-0.04)	0.21 (-0.15–0.40)	0.107
**Enriched in nuclear pore complex interacting proteins**	0.10 (-0.36–0.25)	-0.05 (-0.26–0.31)	0.788
**Erythrocytes**	-0.56 (-0.72–-0.09)	-0.13 (-0.55–0.00)	0.420
**G protein coupled receptors cluster**	0.34 (-0.31–0.78)	0.26 (-0.36–0.46)	0.858
**Inflammation II**	0.53 (0.46–0.66)	0.19 (-0.18–0.78)	0.370
**Inflammation IV**	0.20 (-0.08–0.70)	0.38 (0.21–0.89)	0.654
**Inflammation VI**	0.19 (0.06–0.24)	-0.08 (-0.43–-0.01)	**0.049**
**Inositol phosphate metabolism**	0.17 (-0.06–0.51)	0.34 (0.17–0.45)	0.720
**Interferon**	0.90 (0.51–1.24)	0.40 (0.07–0.90)	0.325
**Intracellular transport**	-0.29 (-0.56–-0.01)	0.06 (-0.22–0.46)	0.210
**Mitochondrial cluster**	-0.89 (-1.15–-0.64)	-0.12 (-0.82–0.23)	**0.049**
**Myeloid cell enriched receptors and transporters**	0.72 (0.44–0.91)	0.30 (-0.28–0.65)	0.370
**Myeloid, dendritic cell activation via NFkB (I)**	-0.71 (-1.26–-0.37)	-0.32 (-0.82–0.04)	0.591
**Plasma cells, immunoglobulins**	0.11 (-0.83–0.67)	0.02 (-0.30–0.57)	0.720
**Platelets**	-0.20 (-0.59–0.14)	-0.10 (-0.61–0.23)	0.929
**Regulation of transcription, transcription factors**	-0.50 (-0.61–-0.24)	-0.05 (-0.18–0.48)	0.152
**Resting dendritic cell surface signature**	-0.57 (-0.89–-0.44)	-0.27 (-0.59–-0.16)	0.282
**Small GTPase mediated signal transduction**	0.35 (-0.06–0.46)	-0.26 (-0.87–0.20)	0.179
**Spliceosome**	-0.71 (-0.92–-0.42)	-0.22 (-0.45–0.43)	0.107
**T cell**	-1.06 (-1.36–-0.81)	-0.22 (-0.74–0.09)	0.152

Data are presented as median (interquartile range). The total number of patients with available data is indicated. All *p*-values are derived from non-parametrical Mann-Whitney *U* tests. Statistically significant *p* values are in bold.

anti-dsDNA: antibodies against double-stranded DNA; CNS: central nervous system; NFkB: Nuclear Factor Kappa B; NK: natural killer.

Supplementary Table S30. Z-scores of gene modules in patients with active CNS lupus and anti-SSA/Ro60 positivity versus negative patients

Gene module	anti-SSA/Ro60 (+) n=5	anti-SSA/Ro60 (-) n=13	*p*-value
**B cell**	-1.05 (-1.12–-0.65)	-0.55 (-1.11–-0.01)	0.218
**Cell cycle**	0.84 (0.49–1.01)	0.18 (-0.05–0.55)	0.104
**Cytotoxic/NK cell**	-0.31 (-1.14–0.01)	-0.25 (-0.95–0.22)	0.805
**Enriched for ubiquitination**	-0.31 (-0.71–0.38)	0.21 (-0.15–0.53)	0.218
**Enriched in nuclear pore complex interacting proteins**	-0.09 (-0.42–0.19)	0.00 (-0.14–0.38)	0.349
**Erythrocytes**	-0.26 (-0.55–-0.13)	-0.07 (-0.61–0.24)	0.460
**G protein coupled receptors cluster**	0.61 (0.06–0.70)	0.01 (-0.53–0.36)	0.218
**Inflammation II**	0.78 (-0.18–0.80)	0.19 (0.07–0.59)	0.460
**Inflammation IV**	0.27 (-0.04–0.51)	0.38 (0.01–0.55)	0.805
**Inflammation VI**	-0.02 (-0.27–0.20)	-0.05 (-0.22–0.15)	0.805
**Inositol phosphate metabolism**	0.33 (0.03–0.34)	0.34 (0.12–0.40)	0.730
**Interferon**	0.68 (0.39–0.76)	0.40 (0.03–0.98)	0.805
**Intracellular transport**	-0.44 (-0.49–0.76)	0.01 (-0.21–0.29)	0.882
**Mitochondrial cluster**	-1.04 (-1.08–0.42)	-0.12 (-0.38–0.19)	0.349
**Myeloid cell enriched receptors and transporters**	0.05 (-0.45–1.03)	0.50 (-0.15–0.64)	0.882
**Myeloid, dendritic cell activation via NFkB (I)**	-0.82 (-1.41–-0.60)	-0.19 (-0.60–0.08)	0.055
**Plasma cells, immunoglobulins**	0.02 (-0.04–0.90)	-0.27 (-0.37–0.49)	0.153
**Platelets**	-0.09 (-0.59–-0.05)	-0.19 (-0.69–0.23)	0.588
**Regulation of transcription, transcription factors**	-0.54 (-0.56–-0.11)	0.17 (-0.18–0.48)	0.068
**Resting dendritic cell surface signature**	-0.60 (-0.67–-0.26)	-0.21 (-0.31–-0.13)	0.183
**Small GTPase mediated signal transduction**	-0.26 (-0.64–0.41)	0.19 (-0.73–0.25)	0.961
**Spliceosome**	-0.45 (-0.61–0.41)	-0.22 (-0.35–0.44)	0.349
**T cell**	-1.09 (-1.32–-0.74)	-0.12 (-0.26–0.10)	**0.043**

Data are presented as median (interquartile range). The total number of patients with available data is indicated. All *p*-values are derived from non-parametrical Mann-Whitney *U* tests. Statistically significant *p* values are in bold.

CNS: central nervous system; NFkB: Nuclear Factor Kappa B; NK: natural killer.

Supplementary Table S31. Z-scores of gene modules in patients with active CNS lupus and low versus patients with normal/high levels of C3c

Gene module	Low C3c n=7	Normal/high C3c n=14	*p*-value
**B cell**	-1.05 (-1.52–-0.60)	-0.67 (-1.37–-0.01)	0.412
**Cell cycle**	0.49 (0.26–1.07)	0.32 (-0.01–0.77)	0.296
**Cytotoxic/NK cell**	-0.63 (-1.14–-0.31)	-0.28 (-1.01–0.17)	0.456
**Enriched for ubiquitination**	-0.15 (-0.29–-0.12)	0.25 (0.01–0.49)	0.062
**Enriched in nuclear pore complex interacting proteins**	0.31 (-0.11–0.48)	-0.07 (-0.25–0.00)	0.179
**Erythrocytes**	0.00 (-0.58–0.43)	-0.25 (-0.58–-0.03)	0.456
**G protein coupled receptors cluster**	0.26 (-0.51–0.85)	0.20 (-0.12–0.44)	0.765
**Inflammation II**	0.80 (0.21–0.85)	0.30 (0.08–0.60)	0.136
**Inflammation IV**	0.01 (-0.09–0.23)	0.53 (0.38–0.95)	**0.006**
**Inflammation VI**	-0.02 (-0.08–0.19)	-0.22 (-0.41–0.06)	0.233
**Inositol phosphate metabolism**	0.34 (0.04–0.41)	0.37 (0.21–0.48)	0.456
**Interferon**	0.68 (0.23–1.05)	0.43 (0.03–0.88)	0.502
**Intracellular transport**	-0.09 (-0.46–0.35)	0.03 (-0.22–0.28)	0.709
**Mitochondrial cluster**	-0.45 (-0.87–-0.20)	-0.22 (-0.79–0.22)	0.456
**Myeloid cell enriched receptors and transporters**	0.55 (0.17–0.76)	0.38 (-0.25–0.91)	0.823
**Myeloid, dendritic cell activation via NFkB (I)**	-0.23 (-0.68–0.14)	-0.64 (-1.26–-0.22)	0.179
**Plasma cells, immunoglobulins**	0.21 (-0.17–0.46)	-0.12 (-0.36–0.59)	0.709
**Platelets**	-0.23 (-0.66–-0.14)	-0.07 (-0.52–0.30)	0.179
**Regulation of transcription, transcription factors**	0.37 (-0.32–0.49)	-0.08 (-0.44–0.16)	0.551
**Resting dendritic cell surface signature**	-0.29 (-0.60–-0.15)	-0.29 (-0.64–-0.17)	0.765
**Small GTPase mediated signal transduction**	0.20 (-0.39–0.47)	-0.38 (-0.98–0.23)	0.101
**Spliceosome**	-0.60 (-0.71–-0.28)	0.35 (-0.40–0.56)	0.052
**T cell**	-0.22 (-0.81–-0.08)	-0.28 (-1.12–0.04)	0.881

Data are presented as median (interquartile range). The total number of patients with available data is indicated. All *p*-values are derived from non-parametrical Mann-Whitney *U* tests. Statistically significant *p* values are in bold.

C3c: complement component 3c; CNS: central nervous system; NFkB: Nuclear Factor Kappa B; NK: natural killer.

Supplementary Table S32. Z-scores of gene modules in patients with active CNS lupus and low versus patients with normal/high levels of C4

Gene module	Low C4<br>n=9	Normal/high C4<br>n=12	*p*-value
**B cell**	-1.05 (-1.72–-0.50)	-0.72 (-1.20–-0.02)	0.477
**Cell cycle**	0.44 (0.15–1.12)	0.32 (0.16–0.62)	0.670
**Cytotoxic/NK cell**	-0.63 (-1.23–-0.45)	-0.21 (-0.60–0.31)	0.136
**Enriched for ubiquitination**	-0.15 (-0.40–0.23)	0.24 (-0.03–0.43)	0.088
**Enriched in nuclear pore complex interacting proteins**	0.21 (-0.23–0.38)	-0.07 (-0.30–0.00)	0.286
**Erythrocytes**	-0.07 (-0.61–0.00)	-0.25 (-0.56–0.06)	0.887
**G protein coupled receptors cluster**	0.26 (-0.36–0.61)	0.21 (-0.22–0.52)	0.943
**Inflammation II**	0.64 (0.47–0.84)	0.16 (0.00–0.52)	0.136
**Inflammation IV**	0.21 (0.00–0.26)	0.47 (0.35–0.90)	0.155
**Inflammation VI**	-0.05 (-0.22–0.20)	-0.15 (-0.38–0.01)	0.320
**Inositol phosphate metabolism**	0.12 (-0.05–0.46)	0.37 (0.32–0.44)	0.434
**Interferon**	0.68 (0.07–1.13)	0.43 (0.09–0.83)	0.434
**Intracellular transport**	-0.21 (-0.49–0.06)	0.21 (-0.18–0.70)	0.076
**Mitochondrial cluster**	-0.84 (-1.04–-0.38)	0.02 (-0.42–0.45)	**0.033**
**Myeloid cell enriched receptors and transporters**	0.30 (-0.15–0.87)	0.54 (0.11–0.75)	0.570
**Myeloid, dendritic cell activation via NFkB (I)**	-0.60 (-0.82–0.04)	-0.46 (-0.97–-0.13)	0.943
**Plasma cells, immunoglobulins**	0.02 (-0.30–0.57)	0.13 (-0.33–0.62)	0.722
**Platelets**	-0.23 (-0.69–-0.05)	-0.10 (-0.38–0.29)	0.320
**Regulation of transcription, transcription factors**	-0.05 (-0.54–0.48)	-0.07 (-0.24–0.21)	0.887
**Resting dendritic cell surface signature**	-0.59 (-0.67–-0.29)	-0.23 (-0.48–-0.07)	0.118
**Small GTPase mediated signal transduction**	0.19 (-0.73–0.45)	-0.38 (-1.00–0.21)	0.155
**Spliceosome**	-0.60 (-0.72–-0.35)	0.41 (-0.23–0.48)	**0.023**
**T cell**	-0.52 (-1.32–-0.12)	-0.20 (-0.82–-0.01)	0.394

Data are presented as median (interquartile range). The total number of patients with available data is indicated. All *p*-values are derived from non-parametrical Mann-Whitney *U* tests. Statistically significant *p* values are in bold.

C4: complement component 4; CNS: central nervous system; NFkB: Nuclear Factor Kappa B; NK: natural killer.

Supplementary Table S33. Z-scores of gene modules in patients with active CNS lupus and PFLC versus patients with no PFLC

Gene module	PFLC n=5	No PFLC n=13	*p*-value
**B cell**	-0.79 (-1.11–-0.50)	-0.65 (-1.32–-0.02)	0.882
**Cell cycle**	0.09 (-0.07–0.18)	0.49 (0.21–1.12)	0.127
**Cytotoxic/NK cell**	-0.49 (-1.14–0.29)	-0.45 (-1.23–0.01)	0.805
**Enriched for ubiquitination**	0.27 (-0.47–0.53)	0.01 (-0.15–0.38)	0.730
**Enriched in nuclear pore complex interacting proteins**	0.00 (0.00–0.19)	-0.02 (-0.26–0.38)	0.961
**Erythrocytes**	-0.51 (-0.62–-0.02)	-0.13 (-0.61–0.24)	0.657
**G protein coupled receptors cluster**	0.36 (0.01–0.61)	0.26 (-0.49–0.46)	0.882
**Inflammation II**	0.19 (0.11–0.42)	0.59 (-0.18–0.80)	0.588
**Inflammation IV**	0.40 (0.38–0.55)	0.24 (0.00–0.51)	0.402
**Inflammation VI**	0.15 (-0.43–0.20)	-0.07 (-0.27–-0.01)	0.588
**Inositol phosphate metabolism**	0.32 (0.03–0.34)	0.34 (0.12–0.46)	0.402
**Interferon**	0.31 (0.12–0.47)	0.40 (0.03–0.98)	0.961
**Intracellular transport**	-0.16 (-0.64–0.29)	0.06 (-0.22–0.46)	0.588
**Mitochondrial cluster**	-0.32 (-0.70–0.54)	-0.12 (-0.82–0.19)	0.730
**Myeloid cell enriched receptors and transporters**	0.25 (-0.28–0.50)	0.64 (0.24–1.01)	0.127
**Myeloid, dendritic cell activation via NFkB (I)**	-0.69 (-0.82–-0.60)	-0.30 (-0.77–0.08)	0.402
**Plasma cells, immunoglobulins**	-0.31 (-0.37–0.02)	0.49 (-0.04–0.69)	0.068
**Platelets**	-0.05 (-0.31–0.28)	-0.19 (-0.63–-0.09)	0.522
**Regulation of transcription, transcription factors**	-0.43 (-0.54–0.17)	-0.05 (-0.18–0.48)	0.522
**Resting dendritic cell surface signature**	-0.46 (-0.55–-0.08)	-0.27 (-0.59–-0.16)	0.961
**Small GTPase mediated signal transduction**	0.25 (-0.98–0.33)	-0.14 (-0.73–0.20)	0.882
**Spliceosome**	0.44 (-0.23–0.61)	-0.22 (-0.60–0.42)	0.183
**T cell**	-0.31 (-1.04–-0.26)	-0.12 (-0.74–0.09)	0.588

Data are presented as median (interquartile range). The total number of patients with available data is indicated. All *p*-values are derived from non-parametrical Mann-Whitney *U* tests. Statistically significant *p* values are in bold.

CNS: central nervous system; NFkB: Nuclear Factor Kappa B; NK: natural killer; PFLC: polyclonal free light chains of kappa and lambda type.

Supplementary Table S34. The most enriched signalling molecule networks in patients with active CNS lupus

Regulator	Motif ID	AUC	NES	Targets	TFs
**B cell gene module**					
TBX2	taipale-NAGGTGTGAWN-TBX2-full	0.077	4.51	12	12
**Cytotoxic/NK cell gene module**					
ELF1	encode-UW.Motif.0046	0.066	4.93	39	3
**Inflammation (II)**					
CEBPE	homer-M00019	0.061	5.71	20	4
**Interferon gene module**					
IRF9	taipale-AWCGAAACCGAAACY-IRF9-full	0.143	18.71	159	6
**Mitochondrial cluster gene module**					
ZBTB33	jaspar-PF0008.1	0.031	5.29	208	1
**Myeloid, dendritic cell activation via NFkB (I) gene module**					
SP1	transfac_pro-M00931	0.058	5.68	51	16
**Resting dendritic cell surface signature gene module**					
ZNF143	factorbook-ZNF143-ext	0.033	4.77	381	1
**T cell gene module**					
ZNF143	jaspar-PF0080.1	0.028	4.04	34	1

Genes in replicated gene modules with a mean |z-score| >0.5 in at least one CNS patient subgroup were imputed in iRegulon through Cytoscape to generate signalling molecule networks and identify their chief regulators. The top chief regulators and enriched motifs are displayed.

AUC: area under the cumulative recovery curve; CNS: central nervous system; NES: normalised enrichment score; NFkB: Nuclear Factor Kappa B; NK: natural killer; TFs: transcription factors.

Supplementary Figure S2. The *TBX2* signalling molecule network and annotated drug targets in patients with active CNS lupus

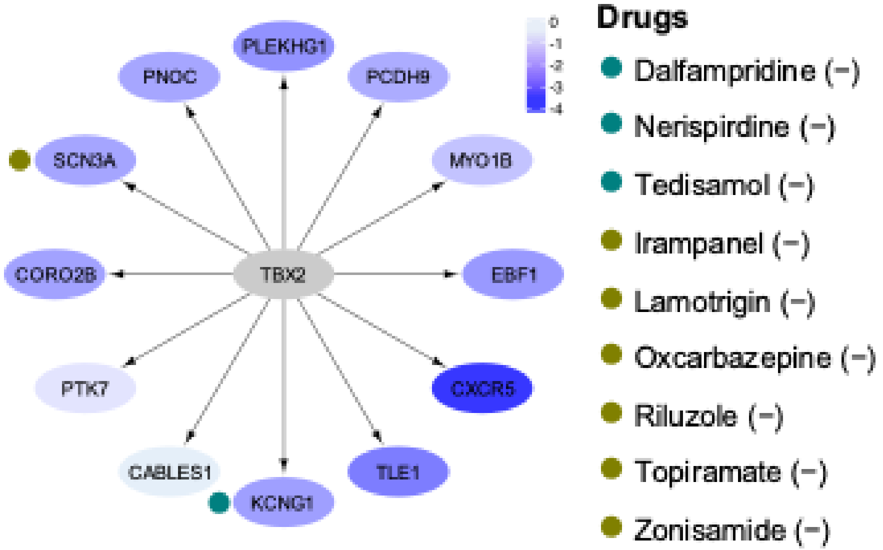

Genes in the B cell gene module were imputed in iRegulon through Cytoscape to generate signalling molecule networks and identify their chief regulators. One of the most enriched signalling molecule networks is plotted, with the chief regulator *TBX2* in the central node. The colour of the nodes ranges from light blue (downregulated genes) to increasing intensities of red (upregulated genes) based on the gene dysregulation (z-scores) in the CNS lupus patient subgroup 1. Coloured dots next to genes indicate drugs modulating these genes, with corresponding drug names listed to the right. Minus and plus signs denote inhibition and stimulation, respectively. Selected drugs of particular relevance are labelled, and the full list of drugs is provided in the Supplementary Material, sheet 10.

CNS: central nervous system; TBX2: T-box transcription factor 2.

Supplementary Figure S3. The *ELF1* signalling molecule network and annotated drug targets in patients with active CNS lupus

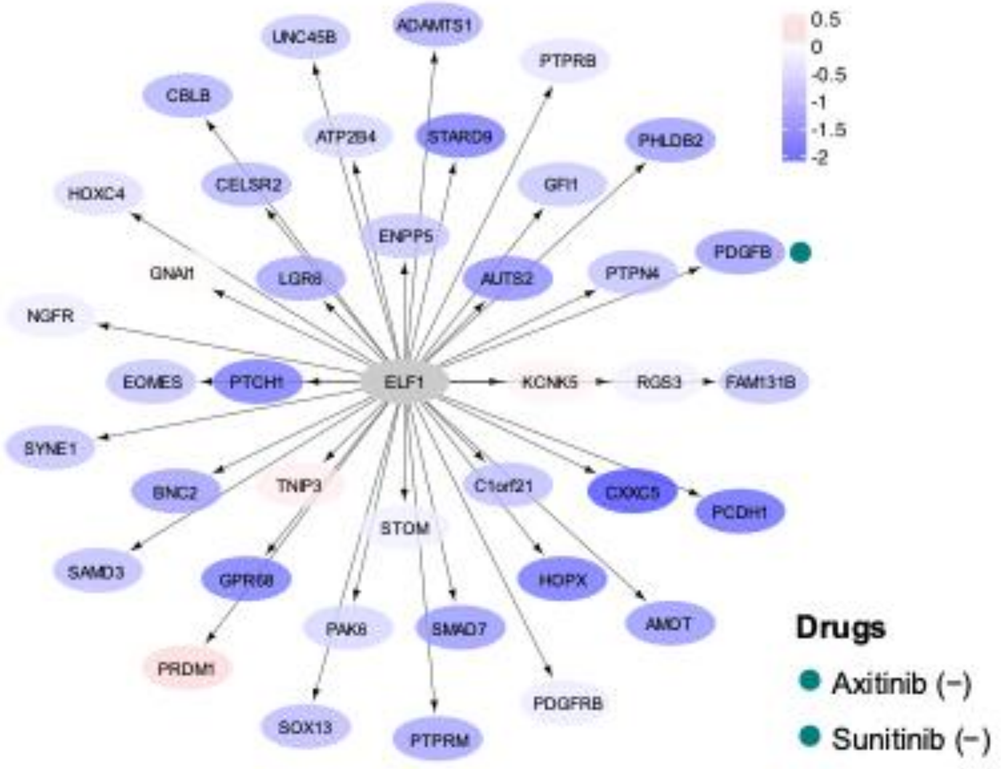

Genes in the cytotoxic/NK cell gene module were imputed in iRegulon through Cytoscape to generate signalling molecule networks and identify their chief regulators. One of the most enriched signalling molecule networks is plotted, with the chief regulator *ELF1* in the central node. The colour of the nodes ranges from light blue (downregulated genes) to increasing intensities of red (upregulated genes) based on the gene dysregulation (z-scores) in the CNS lupus patient subgroup 1. Coloured dots next to genes indicate drugs modulating these genes, with corresponding drug names listed to the right. Minus and plus signs denote inhibition and stimulation, respectively. Selected drugs of particular relevance are labelled, and the full list of drugs is provided in the Supplementary Material, sheet 10.

CNS: central nervous system; ELF1: E74 like ETS transcription factor 1; NK: natural killer.

Supplementary Figure S4. The *CEBPE* signalling molecule network and annotated drug targets in patients with active CNS lupus

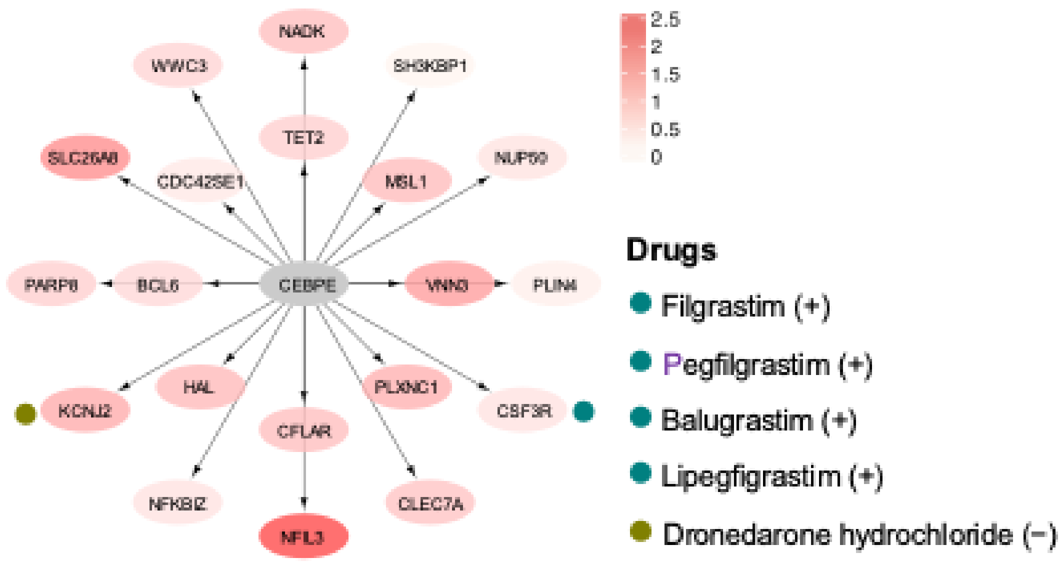

Genes in the inflammation (II) gene module were imputed in iRegulon through Cytoscape to generate signalling molecule networks and identify their chief regulators. One of the most enriched signalling molecule networks is plotted, with the chief regulator *CEBPE* in the central node. The colour of the nodes ranges from light blue (downregulated genes) to increasing intensities of red (upregulated genes) based on the gene dysregulation (z-scores) in the CNS lupus patient subgroup 1. Coloured dots next to genes indicate drugs modulating these genes, with corresponding drug names listed to the right. Minus and plus signs denote inhibition and stimulation, respectively. Selected drugs of particular relevance are labelled, and the full list of drugs is provided in the Supplementary Material, sheet 10.

CEBPE: CCAAT enhancer binding protein epsilon; CNS: central nervous system.

Supplementary Figure S5. The *ZBTB33* signalling molecule network and annotated drug targets in patients with active CNS lupus

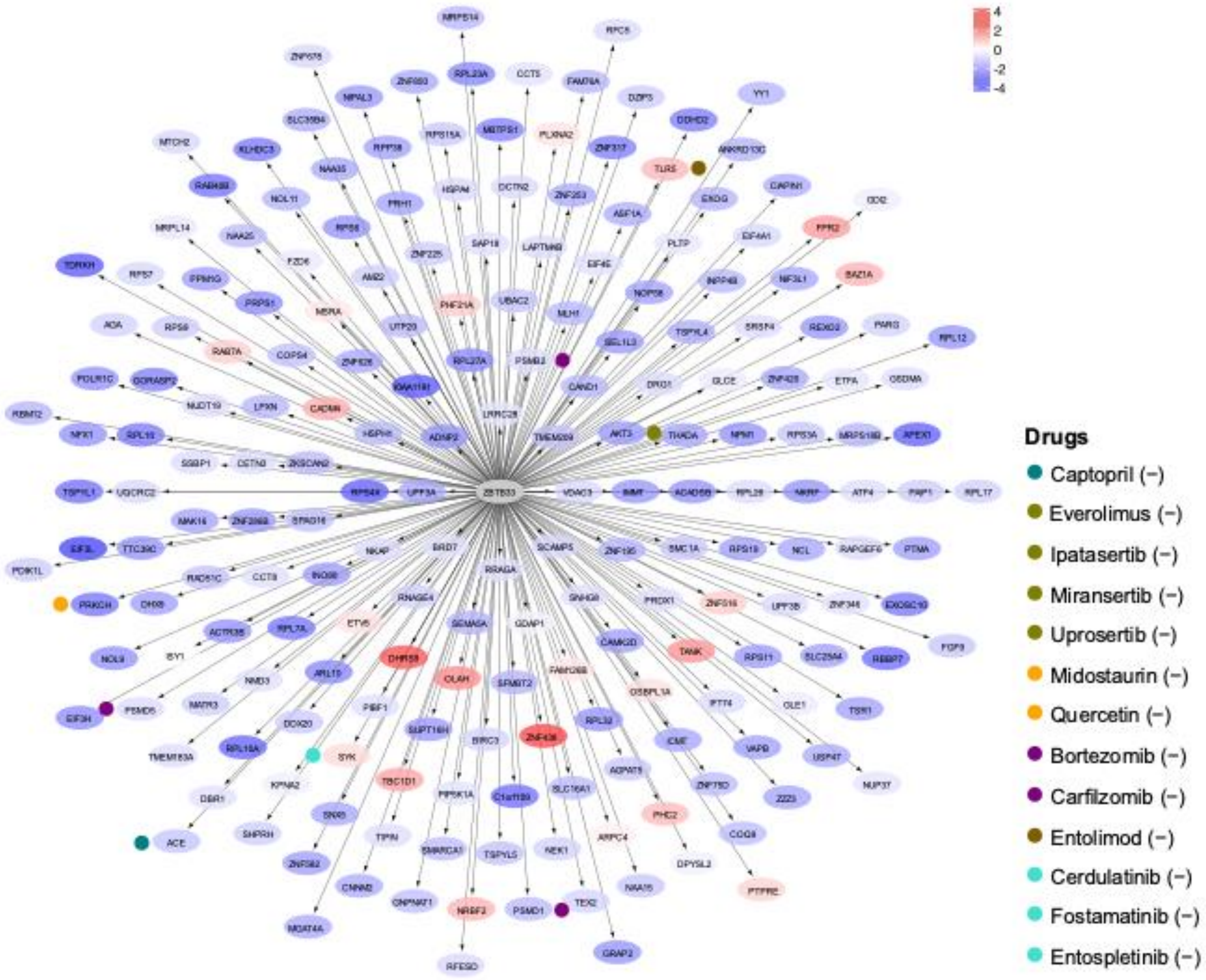

Genes in the mitochondrial cluster gene module were imputed in iRegulon through Cytoscape to generate signalling molecule networks and identify their chief regulators. One of the most enriched signalling molecule networks is plotted, with the chief regulator *ZBTB33* in the central node. The colour of the nodes ranges from light blue (downregulated genes) to increasing intensities of red (upregulated genes) based on the gene dysregulation (z-scores) in the CNS lupus patient subgroup 1. Coloured dots next to genes indicate drugs modulating these genes, with corresponding drug names listed to the right. Minus and plus signs denote inhibition and stimulation, respectively. Selected drugs of particular relevance are labelled, and the full list of drugs is provided in the Supplementary Material, sheet 10.

CNS: central nervous system; ZBTB33: Zinc finger and BTB domain containing 33.

Supplementary Figure S6. The *SP1* signalling molecule network and annotated drug targets in patients with active CNS lupus

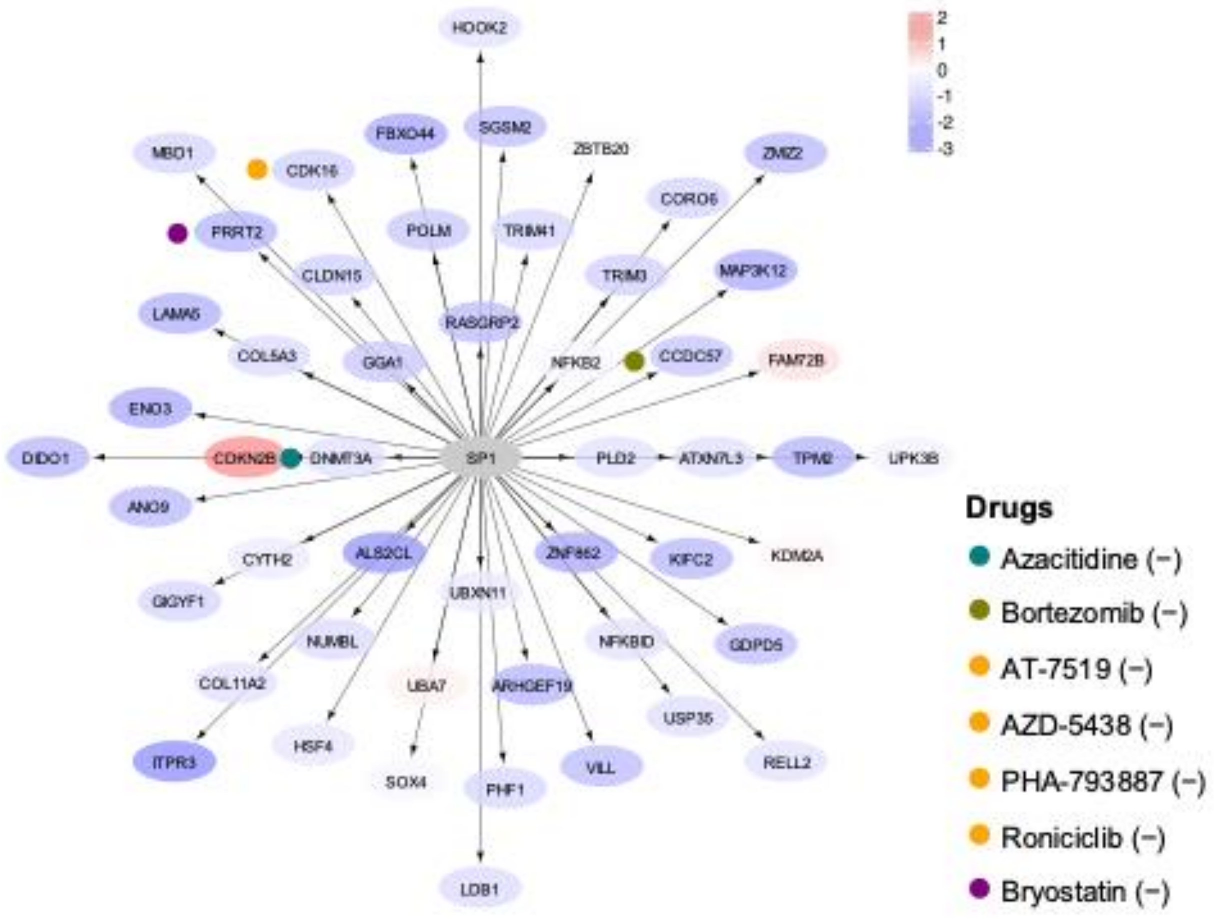

Genes in the myeloid, dendritic cell activation via NFkB (I) gene module were imputed in iRegulon through Cytoscape to generate signalling molecule networks and identify their chief regulators. One of the most enriched signalling molecule networks is plotted, with the chief regulator *SP1* in the central node. The colour of the nodes ranges from light blue (downregulated genes) to increasing intensities of red (upregulated genes) based on the gene dysregulation (z-scores) in the CNS lupus patient subgroup 1. Coloured dots next to genes indicate drugs modulating these genes, with corresponding drug names listed to the right. Minus and plus signs denote inhibition and stimulation, respectively. Selected drugs of particular relevance are labelled, and the full list of drugs is provided in the Supplementary Material, sheet 10.

CNS: central nervous system; NFkB: Nuclear Factor Kappa B.

Supplementary Figure S7. The *ZNF143* signalling molecule network and annotated drug targets in patients with active CNS lupus

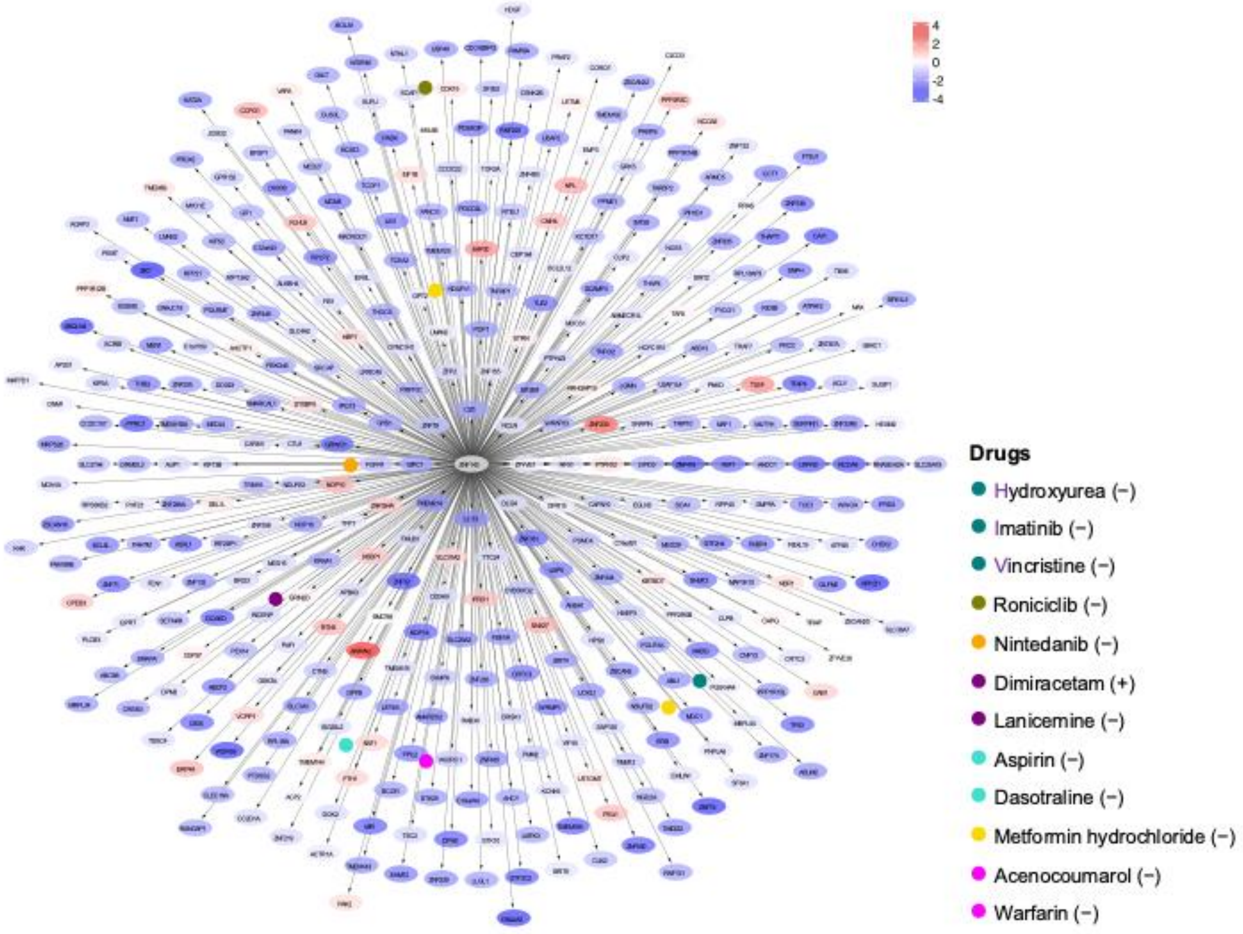

Genes in the resting dendritic cell surface signature gene module were imputed in iRegulon through Cytoscape to generate signalling molecule networks and identify their chief regulators. One of the most enriched signalling molecule networks is plotted, with the chief regulator *ZNF143* in the central node. The colour of the nodes ranges from light blue (downregulated genes) to increasing intensities of red (upregulated genes) based on the gene dysregulation (z-scores) in the CNS lupus patient subgroup 1. Coloured dots next to genes indicate drugs modulating these genes, with corresponding drug names listed to the right. Minus and plus signs denote inhibition and stimulation, respectively. Selected drugs of particular relevance are labelled, and the full list of drugs is provided in the Supplementary Material, sheet 10.

CNS: central nervous system; ZNF143: zinc finger protein 143.

Supplementary Figure S8. The *ZNF143* signalling molecule network and annotated drug targets in patients with active CNS lupus

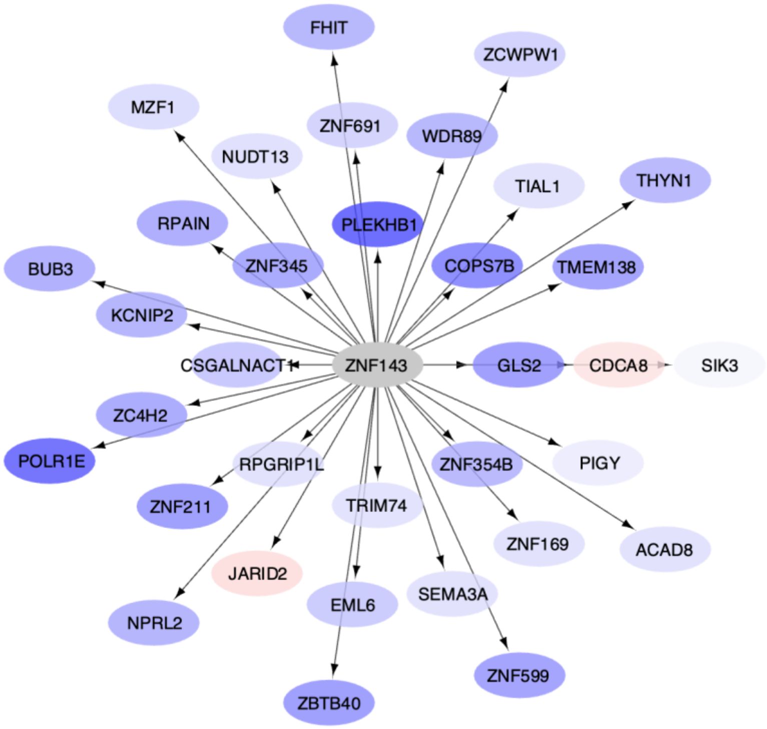

Genes in the T cell gene module were imputed in iRegulon through Cytoscape to generate signalling molecule networks and identify their chief regulators. One of the most enriched signalling molecule networks is plotted, with the chief regulator *ZNF143* in the central node. The colour of the nodes ranges from light blue (downregulated genes) to increasing intensities of red (upregulated genes) based on the gene dysregulation (z-scores) in the CNS lupus patient subgroup 1.

CNS: central nervous system; ZNF143: zinc finger protein 143.

Supplementary Table S35. Response scores to selected targets in patients with active CNS lupus

Target	CNS lupus subgroup 1<br>n=11	CNS lupus subgroup 2<br>n=15	*p*-value
**IFNAR**	0.75±0.05	0.67±0.04	**0.001**
**CD22**	0.10±0.01	0.11±0.01	0.452
**BAFF**	0.30±0.03	0.27±0.02	**0.008**
**BAFF and APRIL**	0.30±0.03	0.27±0.02	**0.008**
**BAFFR**	0.03±0.01	0.04±0.01	0.186
**Calcineurin**	0.07±0.01	0.05±0.01	**<0.001**
**C3**	0.48±0.01	0.42±0.01	**0.002**
**C5**	0.01±0.00	0.01±0.00	0.622
**CD19**	0.27±0.04	0.28±0.04	0.979
**ACE**	0.01±0.00	0.01±0.00	0.697
**PDGFB**	0.29±0.06	0.31±0.05	0.815
**mTORC1**	0.86±0.05	0.86±0.03	0.586
**PRKCH**	0.04±0.01	0.04±0.01	0.421
**TLR5**	0.07±0.01	0.07±0.01	0.139
**SYK**	0.67±0.03	0.63±0.03	**0.002**
**PIK3CA**	0.00±0.00	0.00±0.00	0.123
**CCR1**	0.00±0.00	0.00±0.00	0.243
**CXCL10**	0.17±0.04	0.15±0.03	0.139
**CSF1R**	0.73±0.05	0.70±0.04	0.204

Data are presented as mean (standard deviation). All *p* values are derived from non-parametrical Mann-Whitney *U* tests. Statistically significant *p* values are in bold.

ACE: angiotensin I converting enzyme; APRIL: a proliferation-inducing ligand; BAFF: B cell activating factor belonging to the tumour necrosis factor family; BAFFR; B cell activating factor belonging to the tumour necrosis factor family; C3: complement component 3; C5: complement component 5; CNS: central nervous system; CCR1: CC motif chemokine receptor 1; CSF1R: colony stimulating factor 1 receptor; CXCL10: CXC motif chemokine ligand 10; IFNAR: interferon-α/β receptor; mTORC1: mammalian target of rapamycin complex 1; PDGB: platelet derived growth factor subunit B; PIK3CA: phosphatidylinositol-4,5-bisphosphate 3-kinase catalytic subunit alpha; TLR5: toll-like receptor 5.

Supplementary Table S36. CNS lupus patients with anticipated response to selected drug targets

Target	CNS lupus subgroup 1 n=11	CNS lupus subgroup 2 n=15	*p*-value
**IFNAR**	8 (72.7)	3 (20.0)	**0.015**
**CD22**	5 (45.5)	11 (73.3)	0.228
**BAFF**	7 (63.6)	5 (33.3)	0.257
**BAFF and APRIL**	7 (63.6)	5 (33.3)	0.257
**BAFFR**	5 (45.5)	9 (60.0)	0.736
**Calcineurin**	10 (90.9)	3 (20.0)	**0.001**
**C3**	8 (72.7)	3 (20.0)	**0.015**
**C5**	5 (45.5)	8 (53.3)	1.000
**CD19**	5 (45.5)	8 (53.3)	1.000
**ACE**	5 (45.5)	9 (60.0)	0.736
**PDGFB**	6 (54.5)	8 (53.3)	1.000
**mTORC1**	5 (45.5)	5 (33.3)	0.689
**PRKCH**	4 (36.4)	8 (53.3)	0.646
**TLR5**	4 (36.4)	9 (60.0)	0.427
**SYK**	8 (72.7)	4 (26.7)	0.054
**PIK3CA**	0 (0.0)	2 (13.3)	0.492
**CCR1**	1 (9.1)	0 (0.0)	0.423
**CXCL10**	6 (54.5)	7 (46.7)	1.000
**CSF1R**	7 (63.6)	5 (33.3)	0.257

Data are presented as number (percentage). All *p* values are derived from Pearson's chi squared ($\chi 2$) or Fisher's exact tests. Statistically significant *p* values are in bold.

ACE: angiotensin I converting enzyme; APRIL: a proliferation-inducing ligand; BAFF: B cell activating factor belonging to the tumour necrosis factor family; BAFFR; B cell activating factor belonging to the tumour necrosis factor family; C3: complement component 3; C5: complement component 5; CNS: central nervous system; CCR1: CC motif chemokine receptor 1; CSF1R: colony stimulating factor 1 receptor; CXCL10: CXC motif chemokine ligand 10; IFNAR: interferon-α/β receptor; mTORC1: mammalian target of rapamycin complex 1; PDGB: platelet derived growth factor subunit B; PIK3CA: phosphatidylinositol-4,5-bisphosphate 3-kinase catalytic subunit alpha; TLR5: toll-like receptor 5.

References

[1] M. E. Ritchie, B. Phipson, D. Wu, Y. Hu, C. W. Law, W. Shi *et al.* limma powers differential expression analyses for RNA-sequencing and microarray studies. Nucleic Acids Res, 2015;43**:**e47.

[2] Larsson J. eulerr: Area-Proportional Euler and Venn Diagrams with Ellipses. R package version 7.0.2.

[3] clusterProfiler: an R Package for Comparing Biological Themes Among Gene Clusters. OMICS: A Journal of Integrative Biology, 2012;16**:**284-7.

[4] G. Yu, Q.-Y. He. ReactomePA: an R/Bioconductor package for reactome pathway analysis and visualization. Molecular BioSystems, 2016;12**:**477-9.

[5] M. Ashburner, C. A. Ball, J. A. Blake, D. Botstein, H. Butler, J. M. Cherry *et al.* Gene ontology: tool for the unification of biology. The Gene Ontology Consortium. Nat Genet, 2000;25**:**25-9.

[6] M. Milacic, D. Beavers, P. Conley, C. Gong, M. Gillespie, J. Griss *et al.* The Reactome Pathway Knowledgebase 2024. Nucleic Acids Res, 2024;52**:**D672-D8.

[7] M. Kanehisa, M. Furumichi, M. Tanabe, Y. Sato, K. Morishima. KEGG: new perspectives on genomes, pathways, diseases and drugs. Nucleic Acids Res, 2017;45**:**D353-D61.

[8] D. Chaussabel, C. Quinn, J. Shen, P. Patel, C. Glaser, N. Baldwin *et al.* A modular analysis framework for blood genomics studies: application to systemic lupus erythematosus. Immunity, 2008;29**:**150-64.

[9] S. Li, N. Rouphael, S. Duraisingham, S. Romero-Steiner, S. Presnell, C. Davis *et al.* Molecular signatures of antibody responses derived from a systems biology study of five human vaccines. Nat Immunol, 2014;15**:**195-204.

[10] D. Toro-Domínguez, J. Martorell-Marugán, M. Martinez-Bueno, R. López-Domínguez, E. Carnero-Montoro, G. Barturen *et al.* Scoring personalized molecular portraits identify Systemic Lupus Erythematosus subtypes and predict individualized drug responses, symptomatology and disease progression. Brief Bioinform, 2022.

[11] Kolde R. pheatmap: Pretty Heatmaps. R package version 1.0.12.

[12] C. B. Steen, C. L. Liu, A. A. Alizadeh, A. M. Newman. Profiling Cell Type Abundance and Expression in Bulk Tissues with CIBERSORTx. Methods Mol Biol, 2020;2117**:**135-57.

[13] G. Barturen, S. Babaei, F. Catala-Moll, M. Martinez-Bueno, Z. Makowska, J. Martorell-Marugan *et al.* Integrative Analysis Reveals a Molecular Stratification of Systemic Autoimmune Diseases. Arthritis Rheumatol, 2021;73**:**1073-85.

[14] R. Janky, A. Verfaillie, H. Imrichova, B. Van de Sande, L. Standaert, V. Christiaens *et al.* iRegulon: from a gene list to a gene regulatory network using large motif and track collections. PLoS Comput Biol, 2014;10**:**e1003731.

[15] P. Shannon, A. Markiel, O. Ozier, N. S. Baliga, J. T. Wang, D. Ramage *et al.* Cytoscape: a software environment for integrated models of biomolecular interaction networks. Genome Res, 2003;13**:**2498-504.

[16] A. H. Wagner, A. C. Coffman, B. J. Ainscough, N. C. Spies, Z. L. Skidmore, K. M. Campbell *et al.* DGIdb 2.0: mining clinically relevant drug-gene interactions. Nucleic Acids Res, 2016;44**:**D1036-44.

[17] M. R. Hidalgo, C. Cubuk, A. Amadoz, F. Salavert, J. Carbonell-Caballero, J. Dopazo. High throughput estimation of functional cell activities reveals disease mechanisms and predicts relevant clinical outcomes. Oncotarget, 2017;8**:**5160-78.
